# The Small t Antigen of JC Virus Antagonizes RIG-I-Mediated Innate Immunity by Inhibiting TRIM25’s RNA Binding Ability

**DOI:** 10.1128/mBio.00620-21

**Published:** 2021-04-13

**Authors:** Cindy Chiang, Steve Dvorkin, Jessica J. Chiang, Rachel B. Potter, Michaela U. Gack

**Affiliations:** a Florida Research and Innovation Center, Cleveland Clinic, Port Saint Lucie, Florida, USA; b Department of Microbiology, The University of Chicago, Chicago, Illinois, USA; c Department of Microbiology and Immunobiology, Harvard Medical School, Boston, Massachusetts, USA; Duke University Medical Center

**Keywords:** JC polyomavirus, RIG-I, innate immunity, small t antigen, type I interferon response, viral immune evasion

## Abstract

JC polyomavirus (JCV), a DNA virus that leads to persistent infection in humans, is the causative agent of progressive multifocal leukoencephalopathy, a lethal brain disease that affects immunocompromised individuals. Almost nothing is currently known about how JCV infection is controlled by the innate immune response and, further, whether JCV has evolved mechanisms to antagonize antiviral immunity. Here, we show that the innate immune sensors retinoic acid-inducible gene I (RIG-I) and cGMP-AMP synthase (cGAS) control JCV replication in human astrocytes. We further identify that the small t antigen (tAg) of JCV functions as an interferon (IFN) antagonist by suppressing RIG-I-mediated signal transduction. JCV tAg interacts with the E3 ubiquitin ligase TRIM25, thereby preventing its ability to bind RNA and to induce the K63-linked ubiquitination of RIG-I, which is known to facilitate RIG-I-mediated cytokine responses. Antagonism of RIG-I K63-linked ubiquitination and antiviral signaling is also conserved in the tAg of the related polyomavirus BK virus (BKV). These findings highlight how JCV and BKV manipulate a key innate surveillance pathway, which may stimulate research into designing novel therapies.

## Abstract (precis)

The innate immune response is the first line of defense against viral pathogens, and in turn, many viruses have evolved strategies to evade detection by the host’s innate immune surveillance machinery. Investigation of the interplay between viruses and the innate immune response provides valuable insight into potential therapeutic targets against viral infectious diseases.

## INTRODUCTION

JC virus (JCV) is a member of the *Polyomaviridae* family, which is comprised of small double-stranded DNA viruses and includes BK virus (BKV), simian virus 40 (SV40), and Merkel cell polyomavirus (MCV) (1). The JCV genome is ∼5 kb and encodes the early nonstructural gene products, the large and small t antigens (TAg and tAg, respectively), and the late gene product VP1, VP2, and VP3 capsid proteins, as well as the agnoprotein (2). JCV infects up to 80% of adults and is associated with lifelong, persistent infection in humans (3). In healthy individuals, JCV replicates in the kidney and bone marrow and usually is not associated with any clinical symptoms; however, in immunocompromised patients, JCV can cause progressive multifocal leukoencephalopathy (PML), a potentially lethal brain disease that is characterized by neural demyelination (4). Although the precise mechanisms of how JCV causes pathogenesis still need to be fully determined, it has been shown that JCV can reactivate in immunocompromised individuals, which is accompanied by the appearance of virus in the blood, migration of the neurotropic virus to the central nervous system, and viral entry into astrocytes and oligodendrocytes via clathrin-dependent endocytosis and nonsialylated glycosaminoglycans (3, 5–7). Risk of PML is a serious problem for patients suffering from immunocompromising diseases, such as AIDS, as well as for patients taking certain immunosuppressive drugs for the treatment of autoimmune conditions, like multiple sclerosis (8–10), strengthening the idea that the loss of immune control of JCV infection determines the occurrence of disease.

Recognition of viral pathogens in mammalian cells is mediated by pattern recognition receptors (PRRs), also called innate immune receptors, which surveil the presence of conserved molecular features of the pathogen or recognize host-derived “danger” molecules. PRR activation initiates an innate immune response which limits pathogenesis by activating downstream innate immune mediators as well as the adaptive immune response (11). Four main classes of PRRs have been identified, three of which play major roles in the detection of DNA viruses. These are (i) endosomally localized Toll-like receptor 9 (TLR9), which recognizes viral unmethylated CpG double-stranded DNA (dsDNA) (12); (ii) cyclic GMP (cGMP)-AMP synthase (cGAS) and IFI16, which sense viral dsDNA when it is present in the cytoplasm and/or nucleus (13); and (iii) the retinoic acid-inducible gene I (RIG-I)-like receptors (RLRs), which detect cytosolic dsRNA from RNA viruses and also DNA viruses or noncoding RNAs of host origin (reviewed in detail in reference 14). Whereas TLRs are highly expressed in certain immune cell subtypes, the intracellular receptors RLRs, cGAS, and IFI16 are expressed in virtually all cell types, including epithelial cells, fibroblasts, astrocytes, and neurons.

RLRs are a family of DExD/H box-containing RNA helicases with three members: RIG-I (*DDX58*), MDA5 (melanoma differentiation-associated protein 5; *IFIH1*), and LGP2 (laboratory of genetics and physiology 2) (14). All three RLRs have RNA-binding capacity (mediated by their central helicase and C-terminal domains). However, only RIG-I and MDA5 (not LGP2) can transmit downstream signaling, which is mediated by their N-terminal caspase activation and recruitment domains (CARDs) (15, 16). Binding of RIG-I or MDA5 to immunostimulatory RNA triggers a conformational change and a variety of posttranslational modifications (PTMs) of RLRs. Among the PTMs of RLRs, K63-linked ubiquitination of RIG-I, which triggers its activation, has been studied extensively, and tripartite motif 25 (TRIM25) and other E3 ligases have emerged as important checkpoint regulators for RIG-I signal initiation (17–19). K63-linked ubiquitination of the CARDs of RIG-I was shown to induce oligomerization, while the role of K63-linked ubiquitination for MDA5 CARD-mediated signaling is less well established. RLRs then translocate from the cytosol to mitochondria via 14-3-3ε (RIG-I) or 14-3-3η (MDA5) (20, 21). The CARDs of RIG-I and MDA5 then interact with the CARD of the mitochondrial antiviral signaling protein (MAVS) (22). Binding of the RLRs to MAVS triggers MAVS filament formation, which creates a signaling platform for the recruitment of downstream kinases (i.e., IKK-α/β/γ, TBK1, and/or IKK-ε) and the activation of a variety of transcription factors, most prominently interferon (IFN)-regulatory factors IRF3, IRF5, and IRF7 as well as NF-κB (23). These then trigger antiviral defense mechanisms through upregulation of type I and III IFNs, chemokines, and many other cytokines (14). Cytokines such as type I/III IFNs induce the transcription of a large spectrum of IFN-stimulated genes (ISGs) to directly control virus infection, or they bridge the innate and adaptive arms of the immune response (24).

Not surprisingly, many viruses directly target PRRs, or alternatively, they antagonize PRR-regulatory proteins or downstream signaling molecules as a means to block IFN-mediated innate immune responses (reviewed in references 25 and 26). Furthermore, these so-called viral IFN antagonists can also inhibit signaling by the type I IFN receptor (IFNAR) to impede induction of ISGs. Interestingly, although RLRs are known mainly for their roles in detecting and restricting RNA viruses, recent studies demonstrated that these RNA sensors, and in particular RIG-I, also sense several DNA viruses, such as herpesviruses, poxviruses, and papillomaviruses, by recognizing viral or host-derived RNAs (27–34). As a consequence, not only RNA viruses but also DNA viruses have evolved RLR-inhibitory strategies to promote virus replication and/or establish persistent infection (25, 35).

Currently, the role of innate immunity and specific PRRs in controlling JCV infection is unknown, as is antagonism of the type I IFN response by JCV-encoded proteins. The ability of JCV to maintain persistent infection suggests that the virus may have evolved mechanisms to evade viral detection by the innate surveillance machinery. However, no such mechanisms have been characterized as of yet. Here, we show that both RIG-I and cGAS are critical for controlling JCV infection in human astrocytes. We further identify the JCV tAg as an IFN antagonist that specifically inhibits RIG-I by interfering with its K63-linked polyubiquitination by TRIM25. Finally, we show that antagonism of RIG-I K63-linked ubiquitination is also conserved in the related polyomavirus BKV tAg.

## RESULTS

### The RIG-I and cGAS pathways control JCV replication.

**FIG 1:**
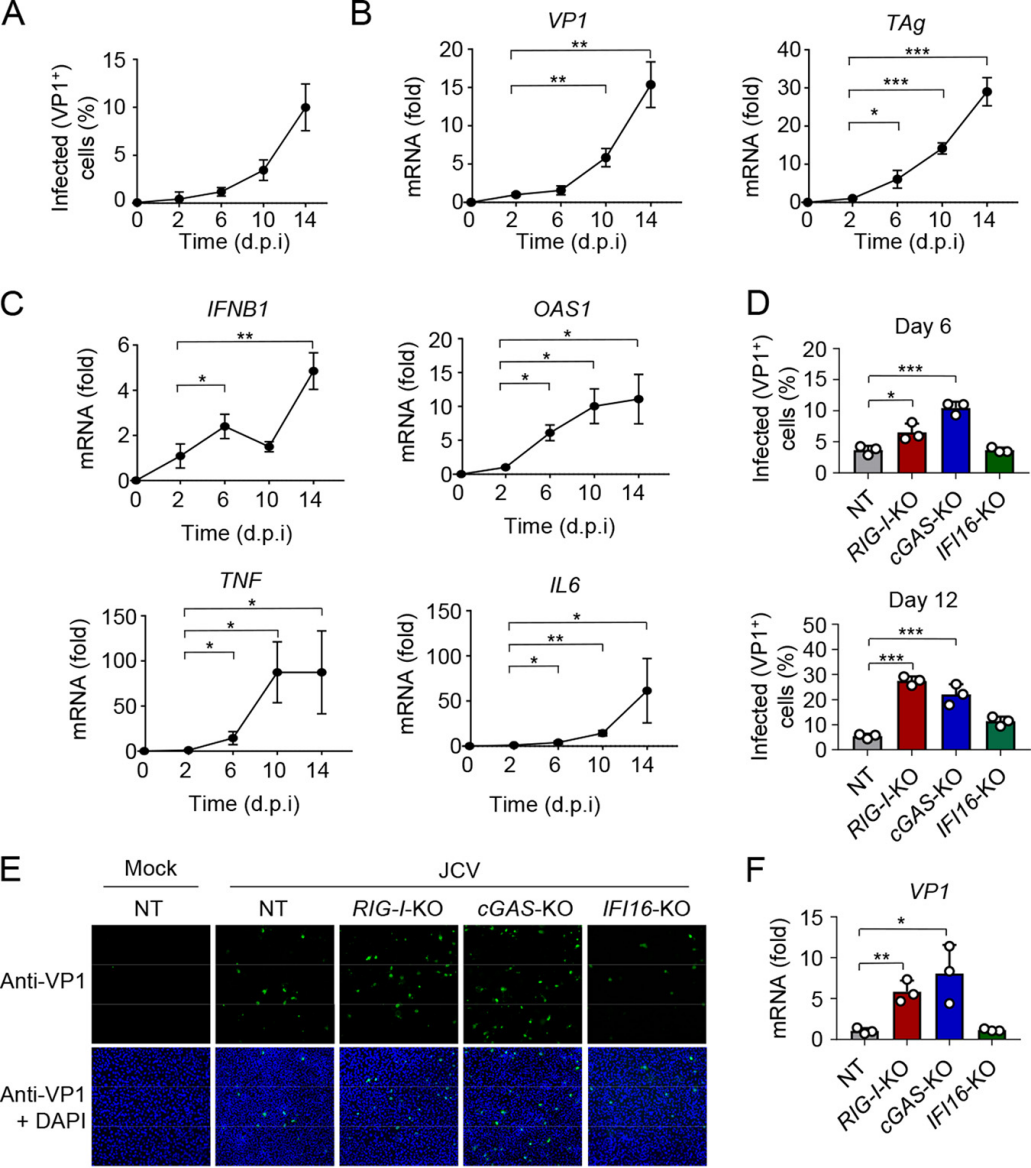
RIG-I and cGAS control JCV replication in human glial astrocyte cells. (A) Frequency of JCV-positive SVGA cells that were infected with JCV (multiplicity of infection [MOI], 0.2) for the indicated times, determined by immunostaining with anti-VP1 and FACS analysis. (B) qRT-PCR analysis of JCV *VP1* (left) and *TAg* (right) transcripts in SVGA cells that were infected with JCV (MOI, 0.2) for the indicated times. (C) qRT-PCR analysis of *IFNB1*, *OAS1*, *TNF*, and *IL6* transcripts in SVGA cells that were infected with JCV (MOI, 0.2) for the indicated times. (D) JCV replication in CRISPR SVGA nontargeting control (NT) cells, or *RIG-I*-KO, *cGAS*-KO, or *IFI16*-KO SVGA cells that were infected with JCV (MOI, 0.2) for 6 (top) or 12 (bottom) days, assessed by immunostaining with anti-VP1 and FACS analysis. (E) JCV infection in CRISPR SVGA NT or KO cells that were infected with JCV (MOI, 0.2) for 6 days, determined by immunostaining with anti-VP1 (green) and confocal microscopy analysis. Mock-infected SVGA NT cells served as an infection specificity control. DAPI was used to stain cell nuclei (blue). (F) qRT-PCR analysis of JCV *VP1* transcripts in CRISPR SVGA NT or indicated KO cells that were infected with JCV (MOI, 0.2) for 12 days. Data are means ± standard deviations (SD) (*n* = 3) and are representative of at least two independent experiments. *, *P* < 0.05; **, *P* < 0.005; ***, *P* < 0.001 (Student's *t* test in panels A, B, C, D, and F). d.p.i, days postinfection.

Work over the past decade demonstrated that most viral pathogens are sensed and restricted by several PRRs of the innate immune system, either in a temporal or in a cell-type specific manner (14, 36). Whereas the sensors responsible for the detection of many human viruses have been discovered, it is currently unknown which intracellular surveillance pathways control JCV infection. Therefore, we sought to investigate whether JCV infection triggers innate immune responses and, further, which innate immune sensors are responsible for controlling JCV replication. To test this, we utilized SVGA human glial astrocytes (37), a commonly used and physiologically relevant target cell type for JCV (38, 39). Importantly, we confirmed that these cells express key innate immune proteins involved in virus sensing: these are the three major intracellular sensors, cGAS, IFI16, and RIG-I, which are known to detect other DNA virus infections (e.g., herpesviruses, papillomaviruses), their respective adaptor proteins (stimulator of IFN genes [STING] and MAVS), and the common downstream mediators TANK binding kinase 1 (TBK1) and ISG15, the latter being a representative ISG (see Fig. S1A in the supplemental material). As in previous studies (38, 40), JCV (Mad-1 strain) productively replicated in SVGA cells over a time course of 14 days, as determined by quantification of JCV VP1 protein or VP1 and TAg transcript expression (Fig. 1A and B). Further, JCV replication was accompanied by a low-level induction of *IFNB1* and *OAS1* (which is an ISG) transcripts (less than 5- and 12-fold, respectively). In contrast, the transcripts of the proinflammatory cytokines interleukin 6 (IL-6) and tumor necrosis factor (TNF) were induced more highly upon JCV infection (up to 50-fold or higher), with greatest induction observed at days 10 to 14 postinfection (Fig. 1C).

10.1128/mBio.00620-21.1 FIG S1 Validation of CRISPR SVGA KO cells and role of MAVS in JCV restriction. (A) Protein abundance of the indicated innate immune proteins in the whole-cell lysates (WCLs) of untreated or IFN-α2-treated (1,000 U/ml for 16 h) SVGA cells, determined by immunoblotting (IB) with the indicated antibodies. WCLs were further probed by IB with antiactin (loading control). (B, upper panel) Endogenous RIG-I and IFI16 protein abundance in the WCLs of CRISPR nontargeting (NT) or the indicated KO SVGA cells, determined by IB with anti-RIG-I or anti-IFI16. WCLs were further probed by IB with antiactin (loading control). (Lower panel) Gene editing of cGAS was confirmed by sequencing of genomic DNA from SVGA *cGAS*-KO cells (or NT control SVGA cells). The gRNA site is indicated in gray. Numbers indicate nucleotides. (C) Functional validation of CRISPR KO SVGA cells by analyzing *CCL5* transcript induction upon transfection of RABV_LE_-RNA (RIG-I agonist; 200 fmol for 16 h) or poly(dA·dT) (dsDNA that stimulates cGAS and IFI16 activation; 30 ng for 16 h) using qRT-PCR. (D) Endogenous MAVS protein abundance in the WCLs of CRISPR NT or *MAVS-*KO SVGA cells, determined by IB with anti-MAVS. WCLs were further probed by IB with antiactin (loading control). (E) Frequency of JCV infection in CRISPR NT or *MAVS*-KO SVGA cells that were infected with JCV (MOI, 0.2) for 12 days, assessed by immunostaining with anti-VP1 and FACS analysis. (F) qRT-PCR analysis of JCV *VP1* transcripts in CRISPR NT or *MAVS-*KO SVGA cells that were infected with JCV (MOI, 0.2) for 12 days. Data are means ± SD (*n* = 3) and areexperiments. *, *P* < 0.05; **, *P* < 0.005; ***, *P* < 0.001 (Student’s *t* test in panels C, E, and F). d.p.i, days postinfection. Download FIG S1, PDF file, 0.4 MB. Copyright © 2021 Chiang et al. 2021 Chiang et al. https://creativecommons.org/licenses/by/4.0/ This content is distributed under the terms of the Creative Commons Attribution 4.0 International license.

Next, to identify the intracellular PRRs that sense and restrict JCV infection, we gene-edited SVGA cells using CRISPR-Cas9 technology to abrogate the expression of RIG-I (gene name, *DDX58*), cGAS (gene name, *MB21D1*), or IFI16 (Fig. S1B). SVGA cells expressing a nontargeting (NT) control guide RNA served as a control. Knockout (KO) of the sensor proteins was confirmed by immunoblotting (IB), or successful gene-editing was confirmed by sequencing of genomic DNA (Fig. S1B). Furthermore, the loss of functionality of the RIG-I, cGAS, or IFI16 signaling pathway in the respective KO cells was confirmed by measuring chemokine induction in response to specific ligands: rabies virus leader sequence (RABV_LE_), which specifically activates RIG-I, or the synthetic dsDNA poly(dA·dT), which is known to trigger cGAS and IFI16 activation (41, 42) (Fig. S1C). Quantification of JCV VP1-positive cells showed that JCV replication was greatly enhanced in *RIG-I*-KO and *cGAS*-KO SVGA cells compared to that of NT control cells, where cGAS predominantly contributed to JCV restriction early during infection (at 6 days postinfection [dpi]), while both cGAS and RIG-I equally contributed to JCV restriction later during infection (at 12 dpi) (Fig. 1D and E). In contrast, knockout of *IFI16* had minimal or no effect on JCV replication. Consistent with these data, VP1 mRNA expression was enhanced in *RIG-I*-KO and *cGAS*-KO SVGA cells, but not in *IFI16*-KO cells, compared to that in NT control cells (Fig. 1F). Furthermore, JCV replication was significantly increased in *MAVS-*KO SVGA cells compared to that in NT control cells (Fig. S1D to F). Together, these data show that cGAS and RIG-I are the major innate immune sensors that control JCV infection in human glial astrocytes.

### The JCV tAg antagonizes the RIG-I-mediated IFN response.

**FIG 2:**
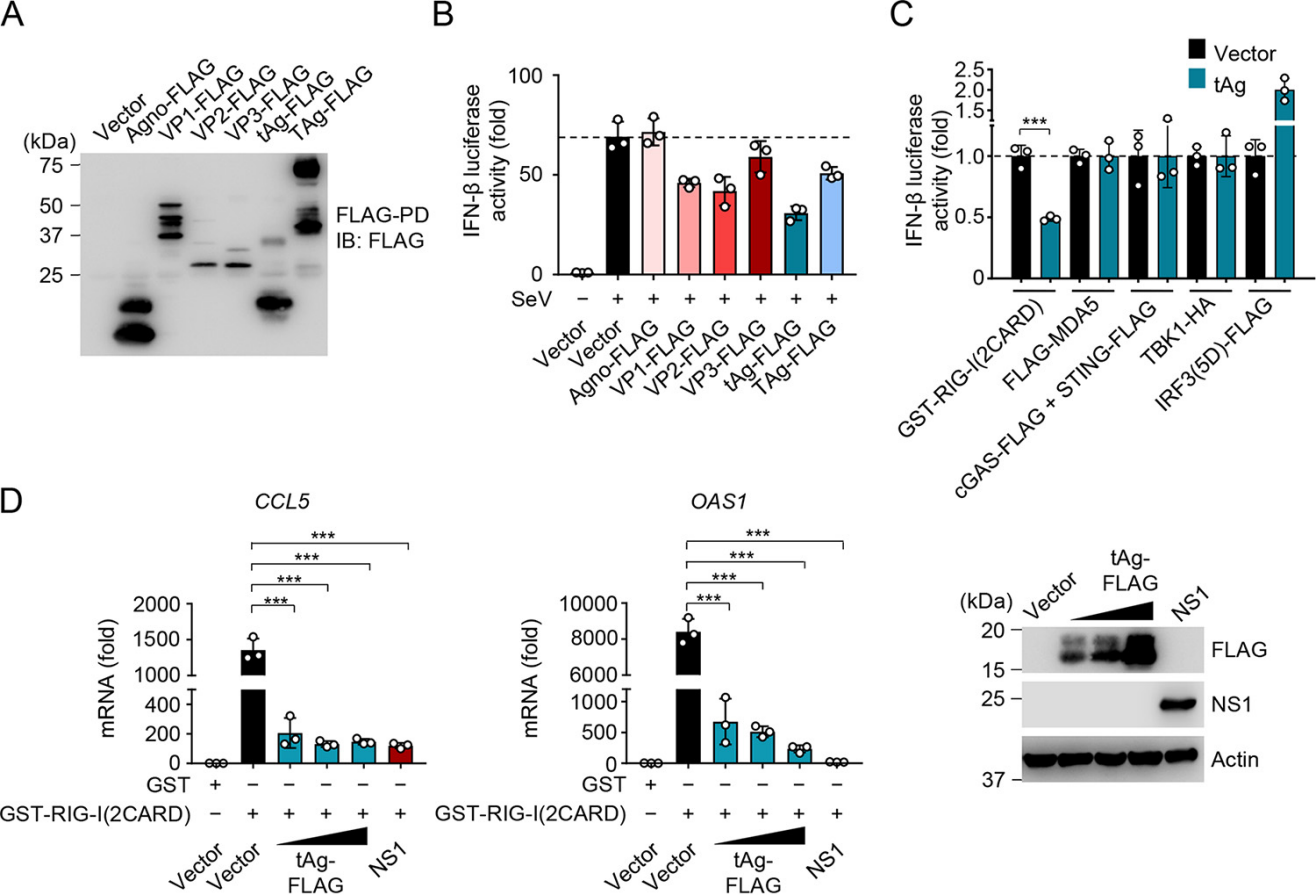
JCV tAg antagonizes RIG-I-mediated innate immune signaling. (A) Representative protein expression of the indicated FLAG-tagged JCV proteins in HEK 293T cells that were transfected for 48 h to express those proteins, determined by FLAG pulldown assay (FLAG-PD) and IB with anti-FLAG. (B) IFN-β luciferase activity in HEK 293T cells that were transfected for 40 h with an empty vector or the indicated FLAG-tagged JCV proteins and then infected with SeV (5 HAU/ml) (which specifically activates RIG-I) for 20 h or left uninfected. Luciferase activity was normalized to values for cotransfected β-galactosidase, and values are presented relative to those for uninfected cells that expressed the empty vector, set to 1. (C) IFN-β luciferase activity induced by transfection of constitutively active GST-RIG-I(2CARD), FLAG-MDA5, cGAS-FLAG and STING-FLAG, TBK1-HA, or the constitutively active mutant of IRF3 [IRF3(5D)-FLAG] with or without cotransfected JCV tAg. Luciferase values were normalized to values for cotransfected β-galactosidase and are presented relative to values for vector-transfected cells, set to 1. (D, left and middle panels) qRT-PCR analysis of *CCL5* or *OAS1* transcripts in HEK 293T cells that were transfected for 40 h with GST or GST-RIG-I(2CARD), together with an empty vector, increasing amounts of FLAG-tagged JCV tAg or IAV NS1 (positive control). (Right) Representative IB confirming protein expression of FLAG-tagged JCV tAg or IAV NS1, determined by IB with anti-FLAG or anti-NS1. IB with antiactin served as a loading control. Data are means ± SD (*n* = 3) and are representative of at least two independent experiments. **, *P* < 0.005; ***, *P* < 0.001 (Student's *t* test in panels B, C, and D). ns, statistically not significant.

Most viruses have evolved effective mechanisms to evade detection by innate immune receptors, which ensures efficient virus replication and establishment of persistent infection. Since our data showed that both cGAS and RIG-I play important roles in restricting JCV infection, we next asked whether JCV-encoded proteins inhibit the signal transduction mediated by these receptors. The JCV genome encodes six viral proteins (2) which have essential functions in the virus life cycle and also modulate a variety of cellular pathways. However, it is currently unknown whether any of the JCV proteins suppress innate immune signaling. To identify JCV proteins that specifically inhibit cGAS- or RIG-I-mediated innate immunity, we tested the effect of ectopic expression of the six individual JCV proteins on IFN-β promoter activation induced by Sendai virus (SeV), which specifically triggers RIG-I-MAVS signaling (Fig. 2A and B), or by cGAS and STING coexpression (Fig. S2A) in a luciferase reporter assay. Unexpectedly, none of the JCV proteins had an appreciable effect on cGAS-STING-mediated IFN-β promoter activation under these conditions (Fig. S2A). However, three JCV proteins showed inhibitory effects on SeV-induced innate immune signaling, where JCV tAg had the strongest suppressive effect, which also was dose dependent (Fig. 2B and Fig. S2B).

10.1128/mBio.00620-21.2 FIG S2 JCV tAg inhibits SeV-induced, but not cGAS-STING- or MAVS-mediated, signaling. (A) IFN-β luciferase activity in HEK 293T cells that were transfected for 40 h with cGAS-FLAG and STING-FLAG together with an empty vector or the indicated FLAG-tagged JCV proteins. Luciferase values were normalized to values for cotransfected β-galactosidase and are presented relative to values for vector-transfected cells, set to 1. (B) IFN-β luciferase activity in HEK 293T cells that were transfected for 40 h with the empty vector, increasing amounts of FLAG-tagged JCV tAg, or IAV NS1 (positive control) and then infected with SeV (10 HAU/ml) for 16 h or left uninfected. Luciferase values were normalized to values for cotransfected β-galactosidase and are presented relative to values for uninfected cells that expressed the empty vector, set to 1. (C) IFN-β luciferase activity in HEK 293T cells that were transfected for 40 h with full-length MAVS (MAVS FL) and either the empty vector or increasing amounts of FLAG-tagged JCV tAg. Luciferase values were normalized to values for cotransfected β-galactosidase and are presented relative to values for vector-transfected cells, set to 1. WCLs were probed by IB with anti-FLAG and antiactin (loading control). (D) Abundances of endogenous ISG15 and ISG54 proteins in the WCLs of HEK 293T cells that were transfected for 24 h with either the empty vector or FLAG-tagged JCV tAg and then mock infected or infected with SeV (50 HAU/ml) for 16 h, determined by IB with anti-ISG15 and anti-ISG54. WCLs were further probed by IB with anti-SeV, anti-FLAG, and antiactin (loading control). Data are means ± SD (*n* = 3) and are representative of at least two independent experiments. *, *P* < 0.05; ** *P* < 0.005; ***, *P* < 0.001 (Student’s *t* test in A, B, and C). ns, statistically not significant. Download FIG S2, PDF file, 0.10 MB. Copyright © 2021 Chiang et al. 2021 Chiang et al. https://creativecommons.org/licenses/by/4.0/ This content is distributed under the terms of the Creative Commons Attribution 4.0 International license.

We also tested the effect of tAg on IFN-β promoter activation triggered by ectopic expression of the related sensor MDA5 or of key downstream proteins of PRR signaling, specifically MAVS, the kinase TBK1, and a constitutively active form of IRF3, IRF3(5D) (43). Whereas JCV tAg effectively blocked the IFN-β promoter activation mediated by RIG-I(2CARD), a constitutively active version of RIG-I, it had no effect on signaling induced by ectopically expressed MDA5, indicating that IFN antagonism by tAg is RIG-I specific. Furthermore, the JCV tAg did not inhibit or only minimally inhibited signaling triggered by ectopic expression of MAVS, TBK1, or IRF3-5D (Fig. 2C and Fig. S2C), suggesting that JCV tAg acts at the level of the sensor RIG-I or perhaps upstream of it.

In support of these data, JCV tAg also suppressed RIG-I(2CARD)-induced transcript induction of *CCL5* and *OAS1* with a potency similar to that of the influenza A virus (IAV) NS1 protein, which is a bona fide viral antagonist of RIG-I signaling and thus served as a positive control (44, 45) (Fig. 2D). The protein abundances of ISG15 and ISG54 induced by SeV infection were also diminished in the presence of JCV tAg compared to those after empty vector transfection (Fig. S2D). Taken together, these results indicate that the JCV tAg functions as an antagonist of IFN induction by specifically targeting the RIG-I signaling pathway.

### TRIM25 is important for control of JCV infection and targeted by JCV tAg.

**FIG 3:**
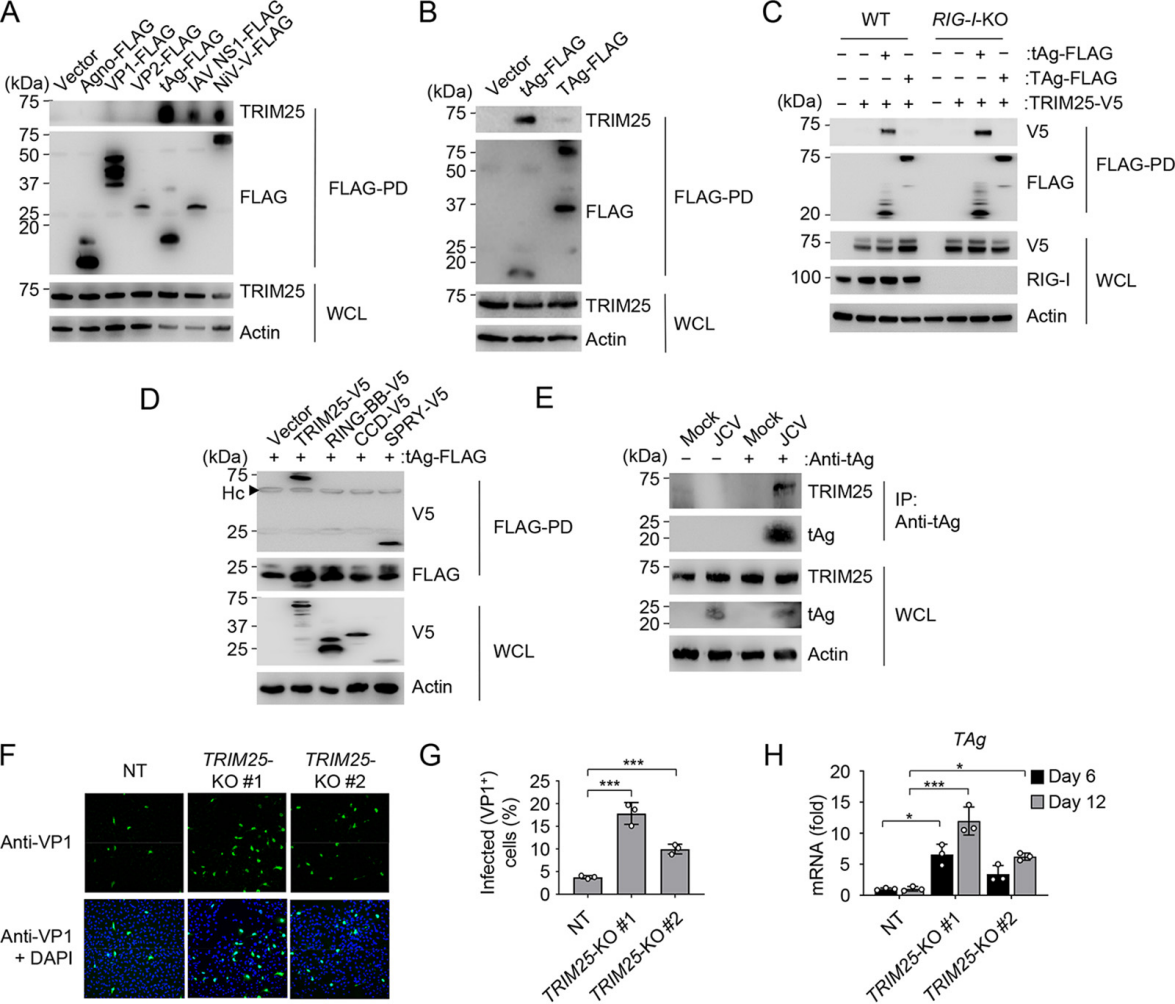
JCV tAg interacts with TRIM25, an E3 ligase that controls JCV infection. (A) Binding of endogenous TRIM25 in HEK 293T cells that were transfected for 48 h with an empty vector, the indicated FLAG-tagged JCV proteins, or the IAV NS1 or NiV V protein (positive controls), determined by FLAG-PD and IB with anti-TRIM25 and anti-FLAG. WCLs were probed by IB with anti-TRIM25 and antiactin (loading control). (B) Binding of endogenous TRIM25 to JCV tAg in HEK 293T cells that were transiently transfected for 48 h with the empty vector or FLAG-tagged JCV tAg or TAg (control), determined by FLAG-PD and IB with anti-TRIM25 and anti-FLAG. WCLs were probed by IB with anti-TRIM25 and antiactin (loading control). (C) Binding of JCV tAg to TRIM25-V5 in WT or *RIG-I*-KO HEK 293T cells that were transfected for 48 h with the vector or TRIM25-V5 together with the empty vector or FLAG-tagged JCV tAg or TAg (control), determined by FLAG-PD and IB with anti-V5 and anti-FLAG. WCLs were probed by IB with anti-V5, anti-RIG-I, and antiactin (loading control). (D) TRIM25-tAg binding assay of HEK 293T cells that were transfected for 48 h with FLAG-tagged JCV tAg together with the empty vector, the V5-tagged TRIM25 full-length (TRIM25-V5) construct, or the indicated TRIM25 truncation constructs, determined by FLAG-PD and IB with anti-V5 and anti-FLAG. WCLs were probed by IB with anti-V5 and antiactin (loading control). RING-BB, RING-finger and B-box domains; CCD, coiled-coil domain; SPRY, SPla/ryanodine receptor (SPRY)/B30.2 domain; Hc, antibody heavy chain. (E) Binding of endogenous TRIM25 to JCV tAg in SVGA cells that were infected with JCV (MOI, 0.2) for 8 days, determined by IP with anti-tAg and IB with anti-TRIM25 and anti-tAg. WCLs were probed by IB with anti-TRIM25, anti-tAg, and antiactin (loading control). Uninfected cells (mock) served as a control. (F) JCV infection in SVGA NT or *TRIM25*-KO cells (nonclonal populations #1 and #2) that were infected with JCV (MOI, 0.2) for 6 days, determined by immunostaining with anti-VP1 (green) and confocal microscopy analysis. DAPI was used to stain cell nuclei (blue). (G) Frequency of JCV infection in CRISPR SVGA NT or *TRIM25*-KO cells (nonclonal cell populations #1 and #2) that were infected with JCV (MOI, 0.2) for 6 days, determined by immunostaining with anti-VP1 and FACS analysis. (H) qRT-PCR analysis of JCV *TAg* transcripts in CRISPR SVGA NT or *TRIM25*-KO cells (nonclonal cell populations #1 and #2) infected with JCV (MOI, 0.2) for 6 or 12 days. Data are means ± SD (*n* = 3) and are representative of at least two independent experiments. *, *P* < 0.05; ***, *P* < 0.001 (Student's *t* test in panels G and H).

We next sought to identify novel interaction partners of the JCV tAg that may provide clues into the molecular mechanism by which tAg antagonizes RIG-I signaling. Mass spectrometry (MS) analysis of the cellular interactome of affinity-purified FLAG-tagged tAg identified with high peptide-coverage TRIM25, an E3 ubiquitin ligase that is known to be critical for RIG-I activation (Fig. S3A). A coimmunoprecipitation (co-IP) assay confirmed an interaction between JCV tAg and human TRIM25. Of note, the binding of tAg to TRIM25 was comparable to or even stronger than that of IAV NS1 and Nipah virus V (NiV-V) protein, which are known to bind and antagonize TRIM25 (44, 46). In contrast to the tAg protein, the JCV Agno, VP1, and VP2 proteins did not bind to TRIM25, strengthening specific TRIM25 targeting by JCV tAg (Fig. 3A). Furthermore, the TAg of JCV, from which tAg is alternatively spliced and shares a conserved DnaJ domain (47), did not bind endogenous TRIM25 either (Fig. 3B). Notably, JCV tAg efficiently bound to exogenous or endogenous TRIM25 in *RIG-I-*KO cells as efficiently as it did in wild-type (WT) control cells, indicating that the tAg-TRIM25 interaction is not mediated by RIG-I and likely has a direct nature (Fig. 3C and Fig. S3B).

10.1128/mBio.00620-21.3 FIG S3 RIG-I is dispensable for tAg-TRIM25 binding, and JCV infection is not controlled by the TRIM25 cofactor ZAP. (A) Unique peptides of TRIM25 identified by mass spectrometry (MS) analysis in complex with FLAG-tagged JCV tAg that was affinity purified from transiently transfected HEK 293T cells. The details of the protein purification and MS are described in Materials and Methods. (B) Binding of JCV tAg to endogenous TRIM25 in WT or *RIG-I-*KO HEK 293T cells that were transfected for 48 h with either an empty vector or FLAG-tagged JCV tAg or TAg (control), determined by FLAG-PD and IB with anti-TRIM25 and anti-FLAG. WCLs were probed by IB with anti-TRIM25, anti-RIG-I, and antiactin (loading control). (C) Validation of SVGA *TRIM25*-KO cells. Abundance of endogenous TRIM25 protein in the WCLs of CRISPR SVGA non-targeting (NT) or *TRIM25*-KO cells (nonclonal populations #1 and #2), determined by IB with anti-TRIM25. WCLs were further probed by IB with antiactin (loading control). (D) Validation of SVGA *ZAP*-KO cells. Endogenous ZAP protein abundance in the WCLs of CRISPR SVGA nontargeting (NT) or *ZAP*-KO cells (nonclonal populations #1 and #2), determined by IB with anti-ZAP. WCLs were further probed by IB with antiactin (loading control). (E) qRT-PCR analysis of JCV *VP1* or *TAg* transcripts in CRISPR SVGA NT, *ZAP*-KO (#1 and #2), or *TRIM25*-KO cells that were infected with JCV (MOI, 0.2) for 6 days. Data are means ± SD (*n* = 3) and are representative of at least two independent experiments. **, *P* < 0.005 (Student’s *t* test). Download FIG S3, PDF file, 0.2 MB. Copyright © 2021 Chiang et al. 2021 Chiang et al. https://creativecommons.org/licenses/by/4.0/ This content is distributed under the terms of the Creative Commons Attribution 4.0 International license.

TRIM25 is characterized by a defined domain structure comprising a central B-box and coiled-coil domain (CCD), which are flanked by an N-terminal RING finger E3 ligase domain (RING) and a C-terminal SPRY (SPla/ryanodine receptor) domain (48). Mapping studies revealed that JCV tAg binds to the TRIM25 SPRY domain, which, despite its low protein expression, efficiently coimmunoprecipitated with JCV tAg. In contrast, the truncation proteins RING-B-box or CCD of TRIM25 did not interact with tAg under the same conditions (Fig. 3D). Importantly, JCV tAg also efficiently bound to endogenous TRIM25 in SVGA cells during native JCV infection (Fig. 3E).

As TRIM25 is a key molecule in the RIG-I signaling pathway (18) and since our data indicated that JCV tAg targets TRIM25, we next determined the relevance of TRIM25 in JCV restriction. To this end, we assessed JCV replication in CRISPR *TRIM25*-KO SVGA cells (two nonclonal populations) (Fig. 3F to H and Fig. S3C). Compared to NT control cells, *TRIM25*-KO SVGA cells showed increased frequency of JCV infection (VP1-positive cells) as well as enhanced viral mRNA expression (Fig. 3F to H). Since TRIM25 not only is involved in RIG-I signaling but, as recently reported, can also activate the zinc finger antiviral protein (ZAP), which suppresses the replication of certain viruses through translational inhibition and/or viral mRNA degradation (49, 50), we tested whether ZAP also controls JCV infection. We observed no significant difference in JCV infection in CRISPR *ZAP*-KO SVGA cells from that in NT control cells (Fig. S3D and S3E), suggesting that TRIM25’s anti-JCV activity is independent of ZAP. Collectively, these results indicate that TRIM25 is important for effective control of JCV replication and that, in turn, JCV tAg targets TRIM25 during infection.

### JCV tAg impedes the ability of TRIM25 to bind RNA and to modify RIG-I with K63-linked polyubiquitin.

**FIG 4:**
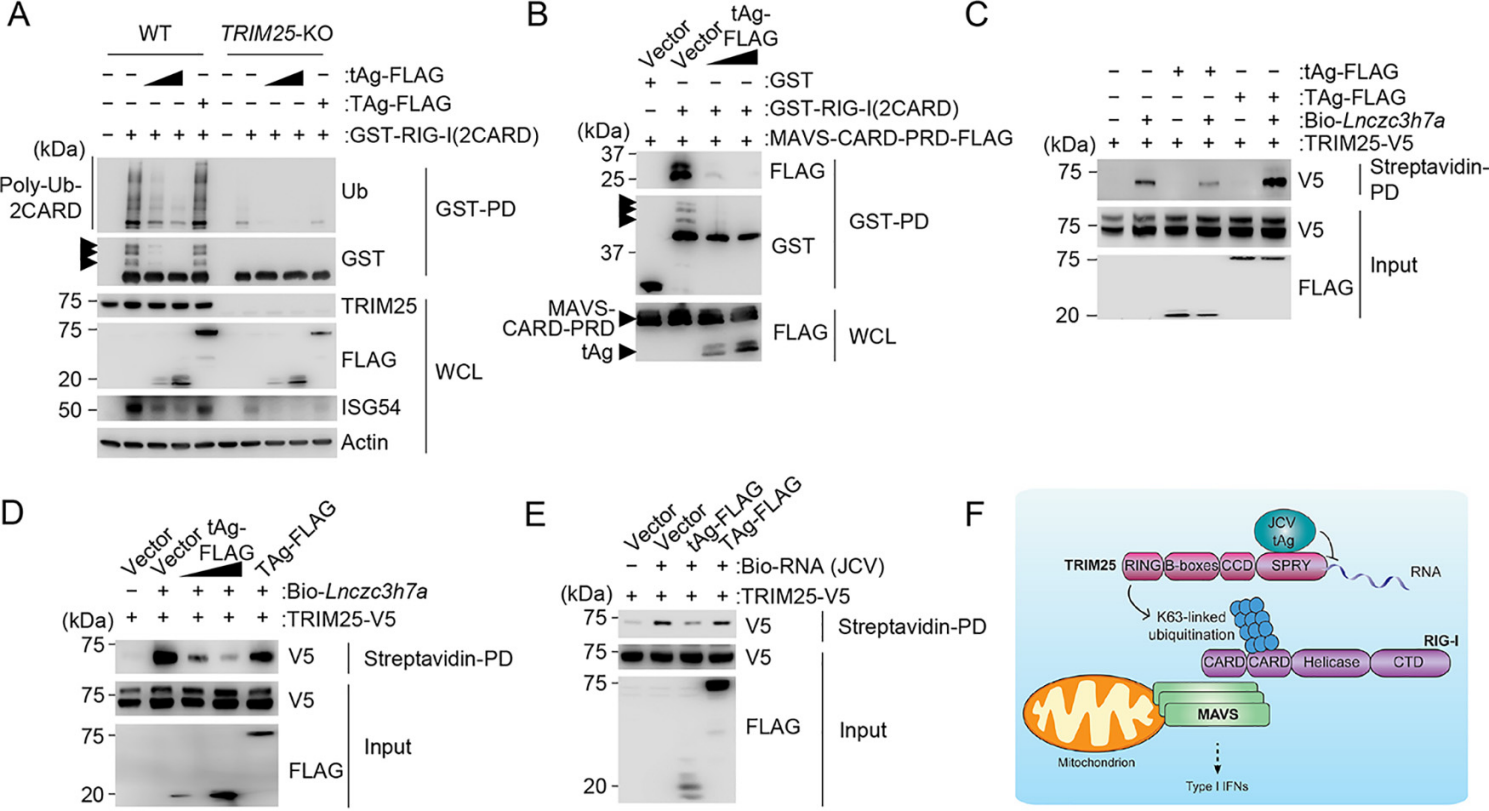
JCV tAg inhibits RNA binding by TRIM25 and blocks the K63-linked ubiquitination of RIG-I. (A) RIG-I CARD ubiquitination in WT or *TRIM25*-KO HEK 293T cells that were transfected for 48 h with GST-RIG-I(2CARD) together with an empty vector or FLAG-tagged tAg or TAg, determined by GST-PD and IB with antiubiquitin (anti-Ub) and anti-GST. WCLs were probed by IB with anti-FLAG, anti-TRIM25, anti-ISG54, and antiactin (loading control). Arrows indicate the ubiquitinated forms of GST-RIG-I(2CARD). (B) Binding of GST-RIG-I(2CARD) or GST (negative control) to FLAG-tagged MAVS-CARD-PRD (proline-rich domain) in the presence or absence of FLAG-tagged JCV tAg in HEK 293T cells that were transfected for 48 h to express those proteins, determined by GST-PD and IB with anti-FLAG and anti-GST. WCLs were probed with anti-FLAG to confirm the expression of MAVS-CARD-PRD and tAg. Arrows indicate the ubiquitinated forms of GST-RIG-I(2CARD). (C) *In vitro* binding of biotinylated *Lnczc3h7a* (Bio-*Lnczc3h7a*) to TRIM25-V5 expressed in HEK 293T cells that were cotransfected with either the empty vector or FLAG-tagged JCV tAg or TAg, determined by streptavidin-pulldown assay (streptavidin-PD) and IB with anti-V5. The protein abundances for TRIM25 and JCV tAg and TAg were determined in the input samples by IB with anti-V5 and anti-FLAG, respectively. (D) *In vitro* binding of biotinylated *Lnczc3h7a* (Bio-*Lnczc3h7a*) to TRIM25-V5 expressed in HEK 293T cells that were cotransfected with either the empty vector, increasing amounts of FLAG-tagged JCV tAg, or FLAG-tagged JCV TAg, determined by streptavidin-PD and IB with anti-V5. The protein abundances for TRIM25 and the JCV proteins tAg and TAg were determined in the input samples by IB with anti-V5 and anti-FLAG, respectively. (E) *In vitro* binding of biotinylated total RNA purified from JCV-infected SVGA cells to TRIM25-V5 expressed in HEK 293T cells that were cotransfected with either the empty vector or FLAG-tagged JCV tAg or TAg, determined by streptavidin-PD and IB with anti-V5. The protein abundances for TRIM25 and the JCV proteins tAg and TAg were determined in the input samples by IB with anti-V5 and anti-FLAG, respectively. (F) Proposed model for the mechanism by which JCV tAg inhibits TRIM25-mediated RIG-I signaling. Data are representative of at least two independent experiments.

We next sought to identify the mechanism by which JCV tAg antagonizes RIG-I signaling. Since tAg effectively binds TRIM25 and because TRIM25 is known to activate RIG-I through catalysis of nondegradative K63-linked polyubiquitination of its N-terminal CARDs, we first tested whether RIG-I ubiquitination was affected by JCV tAg. Ectopic expression of FLAG-tagged JCV tAg profoundly reduced the K63-linked ubiquitination of glutathione *S*-transferase (GST)–RIG-I(2CARD) (Fig. S4A). Furthermore, whereas JCV tAg strongly reduced the K63-linked ubiquitination of RIG-I(2CARD) in WT cells, it had only a small effect on the very low, residual CARD ubiquitination levels in *TRIM25*-KO cells (Fig. 4A). These data confirm that TRIM25 is a major E3 ligase for RIG-I CARD ubiquitination and also strengthen the finding that JCV tAg targets the ubiquitination mediated by TRIM25. As only K63-linked ubiquitinated forms of RIG-I, not the unmodified protein, is known to interact with the adaptor protein MAVS (18, 19), we tested whether the CARD-CARD interaction between RIG-I and MAVS was affected by tAg. We observed a loss of binding between GST–RIG-I(2CARD) and MAVS–CARD–proline-rich domain (MAVS-CARD-PRD) when JCV tAg-FLAG was overexpressed, consistent with a loss of RIG-I(2CARD) ubiquitination (Fig. 4B). In contrast, both proteins interacted efficiently in vector-transfected cells where RIG-I(2CARD) also showed robust K63-linked ubiquitination (Fig. 4B).

10.1128/mBio.00620-21.4 FIG S4 JCV tAg reduces RIG-I(2CARD) ubiquitination but does not block the TRIM25-RIG-I(2CARD) interaction. (A) RIG-I CARD ubiquitination in HEK 293T cells that were transfected for 40 h with GST-RIG-I(2CARD) together with either an empty vector or increasing amounts of FLAG-tagged JCV tAg, determined by GST-PD and IB with anti-GST and anti-Ub. WCLs were probed with anti-FLAG, anti-Ub, and antiactin (loading control). Arrows indicate the ubiquitinated forms of GST-RIG-I(2CARD). (B) Interaction between RIG-I(2CARD) and TRIM25 in HEK 293T cells that were transfected for 40 h with GST (negative control) or GST-RIG-I(2CARD) and TRIM25-V5 together with either the empty vector or increasing amounts of FLAG-tagged JCV tAg, determined by GST-PD and IB with anti-V5 and anti-GST. WCLs were probed by IB with anti-V5 and anti-FLAG. Download FIG S4, PDF file, 0.1 MB. Copyright © 2021 Chiang et al. 2021 Chiang et al. https://creativecommons.org/licenses/by/4.0/ This content is distributed under the terms of the Creative Commons Attribution 4.0 International license.

Since our mapping studies showed that JCV tAg interacts with the C-terminal SPRY domain of TRIM25, we hypothesized that tAg might antagonize specific functions mediated by this domain. The SPRY domain of TRIM25 allows for binding to the RIG-I CARDs (18) and also has recently been shown to have RNA-binding capacity, which ultimately promotes TRIM25 E3 ligase activity to catalyze K63-linked ubiquitination of RIG-I and to induce antiviral signaling (51–53). Although bona fide RNAs that bind and activate TRIM25 in human cells are currently unknown, it was recently discovered that the mouse-specific long noncoding RNA Lnczc3h7a binds to TRIM25, promoting the K63 ubiquitination of RIG-I and thereby innate immunity (52). We therefore tested the ability of JCV tAg to inhibit the RIG-I–TRIM25 interaction and observed only a minor effect of JCV tAg on the binding of GST–RIG-I(2CARD) to TRIM25 (Fig. S4B), suggesting that JCV tAg antagonizes a different function of the TRIM25 SPRY domain.

RNA pulldown assay showed that *in vitro*-transcribed Lnczc3h7a efficiently bound to TRIM25 in the absence of JCV tAg, as previously shown (52), while cotransfected FLAG-tAg abrogated Lnczc3h7a binding to TRIM25 (Fig. 4C). Unlike tAg, FLAG-tagged TAg of JCV, which did not bind to TRIM25 and thus served as a control (Fig. 3B), did not inhibit the Lnczc3h7a-TRIM25 interaction (Fig. 4C). Furthermore, JCV tAg inhibited Lnczc3h7a binding to TRIM25 in a dose-dependent manner (Fig. 4D), suggesting that the viral protein competes with Lnczc3h7a for binding to TRIM25-SPRY. Although Lnczc3h7a is a bona fide TRIM25 RNA agonist, it is mouse specific and not present in human cells. Therefore, we next investigated whether RNA from JCV-infected human cells interacts with TRIM25 and whether JCV tAg inhibits TRIM25’s ability to bind this RNA. Biotinylated total RNA purified from JCV-infected SVGA cells efficiently bound to TRIM25-V5 *in vitro*, while the presence of FLAG-tagged tAg, but not TAg, strongly reduced RNA binding of TRIM25 (Fig. 4E). Taken together, these data indicate that JCV tAg acts as a RIG-I antagonist by binding to its upstream activator, TRIM25. Binding of JCV tAg to the SPRY domain of TRIM25 inhibits its ability to bind RNA, which ultimately prevents TRIM25 from inducing K63 polyubiquitin-mediated RIG-I activation (Fig. 4F).

### BKV tAg blocks RIG-I K63-linked ubiquitination and signaling in a TRIM25-independent manner.

**FIG 5:**
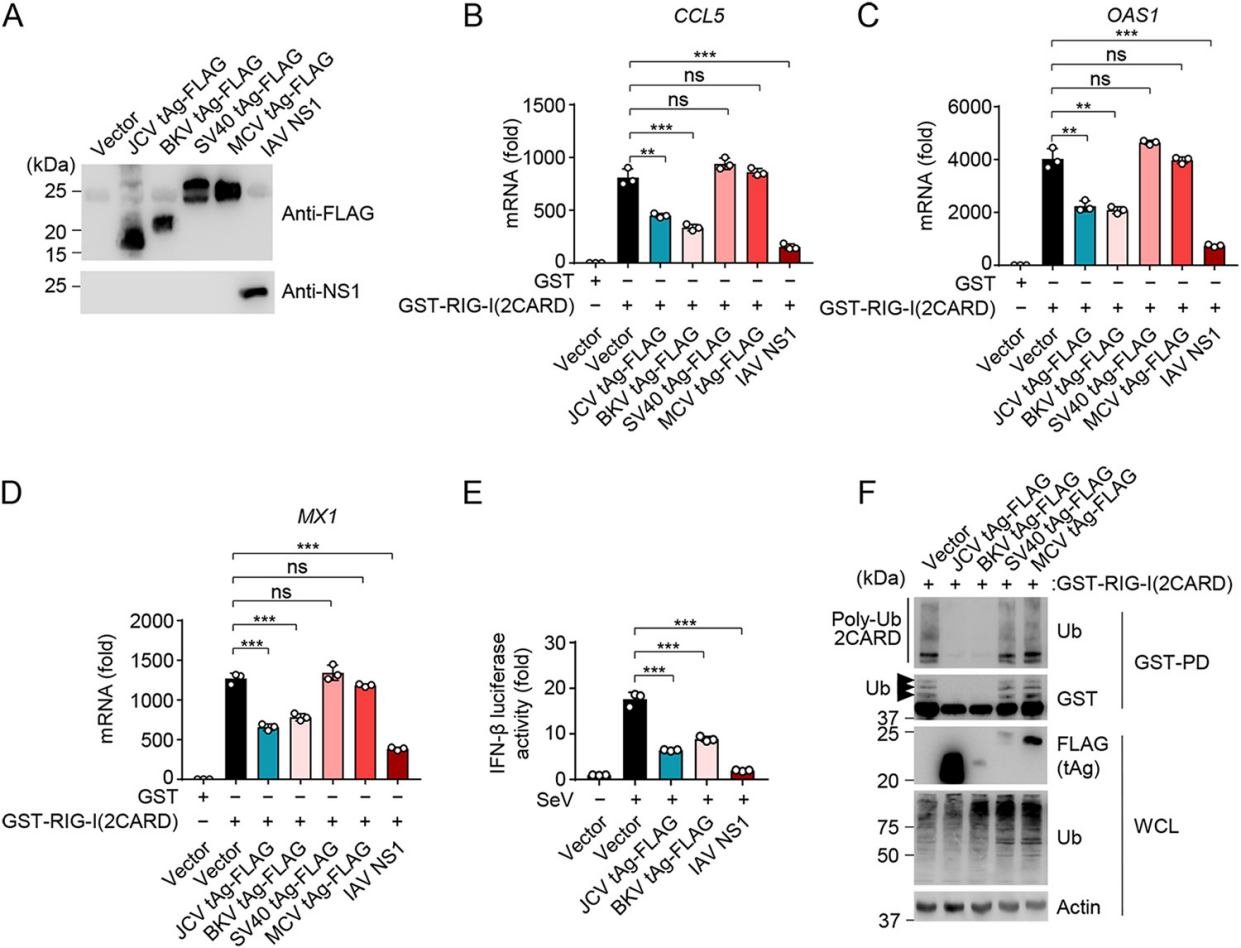
Antagonism of K63-polyubiquitin-dependent RIG-I signaling is also conserved in BKV tAg, but not SV40 or MCV tAg. (A) Representative protein expression of the indicated FLAG-tagged polyomavirus tAgs or untagged IAV NS1 protein in HEK 293T cells that were transiently transfected for 48 h to express those proteins, determined by either FLAG-PD and IB with anti-FLAG, or by IB with anti-NS1. (B) qRT-PCR analysis of *CCL5* transcripts in HEK 293T cells that were transiently transfected for 40 h with either GST-RIG-I(2CARD) or GST (negative control) together with the vector, the indicated FLAG-tagged polyomavirus tAgs, or IAV NS1 (positive control). (C) qRT-PCR analysis of *OAS1* transcripts in HEK 293T cells that were transiently transfected for 40 h with either GST-RIG-I(2CARD) or GST (negative control) together with the vector, the indicated FLAG-tagged polyomavirus tAgs, or IAV NS1 (positive control). (D) qRT-PCR analysis of *MX1* transcripts in HEK 293T cells that were transiently transfected for 40 h with either GST-RIG-I(2CARD) or GST (negative control) together with the vector, the indicated FLAG-tagged polyomavirus tAgs, or IAV NS1 (positive control). (E) IFN-β luciferase activity in HEK 293T cells that were transfected for 40 h with the empty vector, FLAG-tagged JCV tAg or BKV tAg, or IAV NS1 (positive control), and then infected with SeV (10 HAU/ml) for 16 h or left uninfected. Luciferase activity was normalized to values for cotransfected β-galactosidase and are presented relative to values for uninfected cells that expressed the empty vector, set to 1. (F) RIG-I CARD ubiquitination in HEK 293T cells that were transfected for 48 h with GST-RIG-I(2CARD) together with the empty vector or the indicated FLAG-tagged polyomavirus tAgs, determined by GST-PD and IB with anti-Ub and anti-GST. WCLs were further probed by IB with anti-FLAG, anti-Ub, and antiactin (loading control). Arrows indicate the ubiquitinated forms of GST-RIG-I(2CARD). Data are means ± SD (*n* = 3) and are representative of at least two independent experiments. **, *P* < 0.005; ***, *P* < 0.001 (Student's *t* test in panels B, C, D, and E). ns, statistically not significant.

Other viruses in the *Polyomaviridae* family also encode tAg proteins which share significant sequence homology with the JCV tAg. The BKV tAg shares 77% amino acid sequence homology with JCV tAg, while SV40 and MCV tAgs are more distantly related to the tAg of JCV, with 66% and 34% homology, respectively (Fig. S5A). To determine whether RIG-I antagonism is also conserved in tAgs from other polyomaviruses, we compared the effect of FLAG-tagged tAg from BKV, SV40, or MCV on RIG-I(2CARD)-mediated antiviral/proinflammatory gene induction to that of JCV tAg (Fig. 5A to D). In this assay, IAV NS1 was included as an additional control. Similarly to IAV NS1, JCV and BKV tAgs significantly inhibited *CCL5*, *MX1*, and *OAS1* transcript induction by RIG-I(2CARD) compared to that after empty vector cotransfection. In contrast, SV40 and MCV tAgs did not inhibit RIG-I(2CARD)-triggered gene expression. In accord, IFN-β luciferase activity was markedly dampened by ectopic expression of FLAG-tagged JCV and BKV tAg to similar degrees (Fig. 5E). Of note, like JCV tAg, the BKV tAg did not suppress transcriptional responses triggered by ectopic expression of IRF3(5D) (Fig. S5B), ruling out the possibility that BKV tAg acts downstream of RIG-I.

10.1128/mBio.00620-21.5 FIG S5 Polyomaviral tAgs do not inhibit signaling mediated by IRF3. (A) Protein sequence alignment of the tAgs from MCV, SV40, JCV, and BKV generated by Clustal Omega. Numbers indicate amino acids. “*” indicates a fully conserved amino acid, “:” indicates conservation of amino acid groups with similar properties, and “.” indicates amino acids groups with weakly similar properties. (B) qRT-PCR analysis of *IFIT2* transcripts in HEK 293T cells that were transfected for 40 h with IRF3(5D)-FLAG together with the empty vector, the indicated FLAG-tagged polyomavirus tAgs, or IAV NS1. Data are means ± SD (*n* = 3). *, *P* < 0.05 (Student’s *t* test). ns, statistically not significant. (C) Binding of endogenous TRIM25 to polyomavirus tAgs in HEK 293T cells that were transfected for 48 h with the empty vector or the indicated FLAG-tagged polyomavirus tAgs, determined by FLAG-PD and IB with anti-TRIM25 and anti-FLAG. WCLs were probed by IB with anti-TRIM25 and anti-p97 (loading control). Download FIG S5, PDF file, 0.1 MB. Copyright © 2021 Chiang et al. 2021 Chiang et al. https://creativecommons.org/licenses/by/4.0/ This content is distributed under the terms of the Creative Commons Attribution 4.0 International license.

BKV tAg also robustly inhibited RIG-I(2CARD) ubiquitination, similarly to JCV tAg, while SV40 and MCV tAgs had no appreciable effect on 2CARD ubiquitination (Fig. 5F). Of note, although BKV tAg was expressed to much lower levels, it inhibited RIG-I(2CARD) ubiquitination as potently as the JCV tAg, hinting at a distinct mechanism employed by BKV tAg. Indeed, we did not observe an interaction between BKV tAg and endogenous TRIM25 (Fig. S5C), while JCV tAg efficiently bound to TRIM25 under the same conditions, suggesting that BKV tAg inhibits RIG-I signaling in a TRIM25-independent manner. All together, these data show that the tAg of JCV and the closely related BKV suppress RIG-I signaling by blocking the K63-linked ubiquitination of RIG-I; in contrast, SV40 and MCV tAg do not impede RIG-I ubiquitination and signaling. Our data further suggest that BKV tAg inhibits RIG-I CARD ubiquitination using a distinct, TRIM25-independent mechanism.

## DISCUSSION

The role of the innate immune response in controlling persistent JCV infection has not been well studied, nor has the ability of JCV to antagonize specific PRRs and the ensuing type I IFN and ISG induction. JCV infection is known to upregulate IFNs and ISGs in primary human fetal glial cells (54, 55). Furthermore, JCV replication is known to be blocked by type I IFNs in both primary human fetal glial and renal epithelial cells (38, 56). However, it has remained unknown as to which PRR(s) is responsible for the induction of an antiviral response to JCV infection. In this study, we showed, for the first time, that JCV replication in human astrocytes is restricted by the sensors RIG-I and cGAS, supporting the idea that both intracellular RNA and DNA surveillance pathways contribute to the effective control of JCV infection. These findings are in line with recent reports that demonstrated that DNA viruses are sensed by multiple sensors—both DNA sensors (e.g., cGAS and TLR9) and RNA sensors (in particular RIG-I)—that often act in a temporal manner, which is dependent on the emergence of the respective pathogen-associated molecular pattern (PAMP) that they recognize (28, 30–32, 57). For example, herpes simplex virus 1 (HSV-1) is detected in human fibroblasts by cGAS early during infection, while both RIG-I and cGAS equally contribute to innate sensing of HSV-1 late during infection (28). Furthermore, TLR9 detects HSV-1 in bone marrow-derived IFN-producing cells and dendritic cells (58–60). While it is well established that genomic DNA of HSV-1 (and also other DNA viruses) is detected by cGAS, some of the RNA species that are recognized by RIG-I during DNA virus infection have been identified just recently.

Conceptually, during DNA virus infection, RIG-I can recognize virus-derived RNAs or host RNA species whose life cycles are perturbed during DNA virus infection. For instance, during HSV-1 infection, the cellular noncoding RNA *RNA5SP141* acts as a RIG-I agonist, and activation of RIG-I by *RNA5SP141* likely requires remodeling of the nuclear envelope caused by herpesvirus replication in order to relocalize this pseudogene transcript to the cytoplasm, and also host shutoff of protein synthesis. Specifically, virus-induced host shutoff mechanisms prevent the synthesis of specific *RNA5SP141*-binding proteins, which normally shield these noncoding RNAs in the cytoplasm. Downregulation of these shielding proteins during HSV-1 infection then liberates *RNA5SP141*, leading to RIG-I activation (28). For Epstein-Barr virus (EBV) infection, both *RNA5SP141* and viral RNAs (i.e., EBV-encoded small RNAs [EBERs], which are abundantly expressed, small noncoding RNAs) were reported to trigger RIG-I signaling (27, 29, 61). Similarly, during KSHV infection, both viral RNAs and the noncoding polymerase III (Pol III) transcript vault RNAs (vtRNAs) activate the RIG-I-mediated antiviral response (32, 62). Currently, the physiological PAMPs that trigger RIG-I activation during JCV infection are unknown. It remains to be determined whether RIG-I detects viral RNAs produced during JCV infection or whether host RNAs that are unmasked or mislocalized during JCV infection are sensed by RIG-I. Since polyomaviruses are known to employ effective host shutoff mechanisms, which are most well characterized for SV40, whose tAg has been shown to block host mRNA translation through hypophosphorylation of the eIF4e-binding protein 1 (63), it is tempting to speculate that host shutoff strategies employed by JCV may also induce unmasking of *RNA5SP141* or other cellular RNAs, which remains to be determined. Moreover, it is conceivable that viral microRNAs (miRNAs), which are known to be produced during polyomavirus infection (64, 65), serve as potential RIG-I ligands.

Practically all viral pathogens are equipped with effective strategies to block IFN induction or the antiviral mechanisms exerted by type I IFNs, and IFN-antagonistic proteins have been identified for many viruses that cause disease in humans. In striking contrast, to date, no type I IFN antagonist has been identified for JCV. Our work identified the JCV tAg as a type I IFN antagonist that specifically targets the RIG-I signaling pathway. Since polyomavirus tAgs have been most well characterized in their ability to bind to the cellular protein phosphatase 2A (66, 67) or for their oncogenic properties (however, not in the case of JCV tAg) (68, 69), our study unveiled a novel function for polyomaviral tAg proteins that may play a major role for virus persistence and disease outcomes.

Given the important role of TRIM25 in the RIG-I antiviral response, several viruses have evolved to inhibit TRIM25, although through different mechanisms. The NS1 proteins of several IAV strains bind to the CCD of TRIM25, which can improperly position the SPRY domain and interferes with its E3 ligase ability (44, 70). The N protein of severe acute respiratory syndrome coronavirus (SARS-CoV) binds to the SPRY domain of TRIM25 and competitively inhibits binding of the E3 ligase to RIG-I (71). In contrast, paramyxovirus V proteins are able to bind the SPRY domain of TRIM25 as well as the CARD domains of RIG-I and block RIG-I ubiquitination in a noncompetitive manner (46). EBV encodes a viral deconjugase, BPLF1, which binds to the TRIM25–RIG-I complex and counteracts the E3 ligase activity of TRIM25 by removing conjugated ubiquitin as opposed to disrupting TRIM25 function (72). Our work discovered that JCV tAg employs a novel mechanism to antagonize TRIM25, that is, by preventing its ability to bind cellular RNA, which has recently been demonstrated to be important for TRIM25 E3 ligase activity and thereby RIG-I activation (51–53). Consistent with this concept, our data showed that JCV tAg, via binding to the SPRY domain, blocks TRIM25’s RNA binding, which ultimately suppresses RIG-I K63-linked ubiquitination and antiviral signaling. Our data demonstrate that JCV tAg targets TRIM25; however, whether it also affects the activity of other E3 ligases that ubiquitinate RIG-I remains to be elucidated.

As *Lnczc3h7a*, which was recently shown to bind to TRIM25 and to promote RIG-I-mediated antiviral signaling, is a mouse-specific long noncoding RNA (lncRNA) (52), we postulate that a specific human lncRNA exists whose binding to TRIM25 is inhibited by JCV tAg to efficiently antagonize type I IFN responses. Although we show that RNA from JCV-infected astrocytes binds to TRIM25, further studies will be required to identify and characterize the specific RNA species that bind(s) to and activate(s) TRIM25 during JCV infection. Whether this RNA is host or virus derived also remains to be determined. Moreover, we expect that our work will stimulate future studies to determine the relevance of the type I IFN response and viral antagonism thereof for the ability of JCV to establish persistent infection and cause disease in appropriate mouse models and in humans. Along these lines, recently developed humanized mouse models in which mice are engrafted with human thymus or human glial cells (39, 73) may present opportunities to study the role of JCV tAg-mediated RIG-I inhibition in the maintenance of persistent infection in the kidney, JCV neurotropism, and pathogenesis of PML.

We also found that tAg of the most closely related polyomavirus tested, BKV, also suppressed the K63-linked ubiquitination of RIG-I and RIG-I-mediated upregulation of cytokines, chemokines, and ISGs, while SV40 and MCV tAgs had no discernible effect on RIG-I signaling. Interestingly, our data suggest that although antagonism of RIG-I K63 polyubiquitination is conserved in BKV tAg, the mechanism of inhibition is not. In contrast to JCV tAg, binding of BKV tAg to TRIM25 was not detectable by co-IP, suggesting that BKV tAg blocks RIG-I ubiquitination through a yet-to-be-determined mechanism. One possibility is that BKV tAg recruits a host deubiquitinating enzyme (DUB) to inhibit RIG-I K63-linked ubiquitination and its antiviral signaling. The K63-polyubiquitin-dependent activation of RIG-I is reversed by at least three deubiquitinating enzymes, in particular USP3, USP21, and CYLD (74–77), to dampen RIG-I signaling as part of a regulatory circuit. As of yet, no virus has been shown to directly recruit any of these DUBs to antagonize RIG-I signaling. Alternatively, miRNAs produced during BKV infection may regulate the expression of TRIM25 or DUBs, which may ultimately suppress RIG-I ubiquitination. Interestingly, such a mechanism has been described for the 3C protein of enterovirus 71 (EV71), which modulates the expression of CYLD by downregulating host miRNA miR-526a (78). Lastly, another possibility is that BKV tAg may inhibit another E3 ligase (e.g., TRIM4, Riplet, or MEX3C) that reportedly can also catalyze RIG-I CARD ubiquitination (17).

Taking these findings together, we discovered that JCV infection is effectively sensed by the RNA sensor RIG-I; however, JCV has evolved to subvert RIG-I-mediated innate immunity. Our work identified the tAg-TRIM25 interaction as a potential molecular target to impede persistent JCV infection and JCV-associated severe disease (i.e., PML) for which there is currently no specific treatment. Our discovery of the role of cGAS and RIG-I in controlling JCV infection may also stimulate investigations to test specific agonists of these two sensors as potential therapeutics to diminish JCV reactivation or active replication.

## MATERIALS AND METHODS

### Cell culture and transfection.

HEK 293T cells (embryonic, female, human) (ATCC) were cultured in Dulbecco’s modified Eagle’s medium (DMEM; ThermoFisher Scientific) supplemented with 10% (vol/vol) heat-inactivated fetal bovine serum (FBS; Gibco), 2 mM l-glutamine (Gibco), and 1% (vol/vol) penicillin-streptomycin (Gibco) under standard culture conditions. SVGA cells (fetal, human) (provided by E. Cahir-McFarland, Biogen [37]) were cultured in Eagle’s minimum essential medium (MEM; ThermoFisher Scientific) supplemented with 10% (vol/vol) heat-inactivated FBS (Gibco), 2 mM l-glutamine (Gibco), and 1% (vol/vol) penicillin-streptomycin (Gibco) under standard culture conditions. SVGA CRISPR KO cells were cultured in MEM supplemented with 1 μg/ml puromycin. Transient transfections were performed with Lipofectamine and Plus reagent (Life Technologies), with polyethyleneimine (PEI) or with calcium phosphate (Clontech) according to the manufacturer’s instructions.

### Generation of CRISPR KO SVGA and HEK 293T cells.

To generate knockout SVGA cell lines, specific guide RNAs (gRNAs) directed against sequences near the start codon of the gene of interest were designed using the CRISPR MIT tool (Zhang lab, MIT). Briefly, nontargeting (NT) gRNA (5′-gtggaaaggacgaaacaccgACGGAGGCTAAGCGTCGCAAgttttagagctagaaatag-3′), RIG-I gRNA (5′-gtggaaaggacgaaacaccGGGTCTTCCGGATATAATCCgttttagagctagaaatag-3′), cGAS (5′-gtggaaaggacgaaacaccGGGCCGAACTTTCCCGCCTTgttttagagctagaaatag-3′), IFI16 (5′-gtggaaaggacgaaacaccGAAGAACATTGTTCTACTAAAgttttagagctagaaatag-3′), MAVS (5′-gtggaaaggacgaaacaccGAGGTGGCCCGCAGTCGATCCgttttagagctagaaatag-3′), TRIM25 (5′-gtggaaaggacgaaacaccGTCGCGCCTGGTAGACGGCGgttttagagctagaaatag-3′), and 5′-gtggaaaggacgaaacaccGAGCCGGTCACCACTCCGTGgttttagagctagaaatag-3′) or ZAP gRNA (5′-gtggaaaggacgaaacaccGCACAGCCAGCGCGCCATGG gttttagagctagaaatag-3′ and 5′-gtggaaaggacgaaacaccGCAAAATCCTGTGCGCCCACGgttttagagctagaaatag-3′) containing 5′ and 3′ overhangs of the pSicoR-CRISPR-PuroR vector (in lowercase letters) were cloned into the cut pSicoR-CRISPR-PuroR vector at the BsmBI restriction site using Gibson assembly. Briefly, the digested vector, gRNA oligonucleotide, and Gibson master mix (NEB Biolabs; E2611) were incubated at 50°C for 4 h, followed by transformation into competent E. coli TOP10 cells (ThermoFisher Scientific). Plasmids were sequenced to confirm correct insertion of gRNAs, transfected with third-generation lentivirus packaging plasmids, and selected and validated as previously described (33). Whole-cell lysates (WCLs) of CRISPR KO SVGA cells were subjected to IB to confirm the loss of target protein expression. To confirm correct gene editing in *cGAS*-KO SVGA cells, genomic DNA was extracted using the E.Z.N.A. tissue DNA kit (Omega Bio-tek) and then subjected to genomic DNA sequencing. Additionally, SVGA KO cells were validated using functional assays (as described in the supplemental material). *RIG-I-*KO HEK 293T cells were generated using the same RIG-I-specific guide RNA as described above for *RIG-I-*KO SVGA cells. *TRIM25-*KO HEK 293T cells were previously described (79).

### Viruses.

The JCV Mad-1 strain was kindly provided by Ellen Cahir-McFarland (Biogen, Cambridge, MA). JCV was amplified in 293FT cells grown in infection medium (DMEM supplemented with 10% [vol/vol] heat-inactivated FBS [Gibco], 2 mM l-glutamine [Gibco], 1% [vol/vol] penicillin-streptomycin [Gibco], 1× nonessential amino acids [Gibco], and 1 mM sodium pyruvate [Gibco]) in HYPERFlasks (Corning) under standard culture conditions for 2 weeks. Infected cells were harvested and centrifuged at 12,000 × *g* for 30 min at 4°C. The supernatant was removed, and 10% of the supernatant was used to resuspend the cell pellets, which were frozen and thawed three times. Twenty-five units of Benzonase nuclease (Sigma)/ml and 20 U/ml of α2-3,6,8 neuraminidase (New England BioLabs) were added to the cell suspension and incubated overnight in a water bath at 37°C. The cell suspension was centrifuged at 12,000 × *g* for 30 min at 4°C, and the supernatant was combined with the supernatant from the initial centrifugation. Samples were ultracentrifuged over a 40% sucrose layer for 3 h at a 100,000 relative centrifugal force (rcf) at 4°C. Pellets were resuspended in phosphate-buffered saline (PBS), and the virus was tested for infectivity by immunostaining for tAg and flow cytometry.

SeV (Cantell strain) was obtained from Charles River Laboratories.

### Plasmids and reagents.

pDONR223-JCV Agno, VP1, VP2, VP3, tAg, and TAg, as well as pDONR223-BKV tAg, were kindly provided by Ellen Cahir-McFarland (Biogen, Cambridge, MA). The plasmids encoding SV40 tAg and MCV tAg were obtained from Addgene (catalog no. 37858 and 37861, respectively). All were subcloned into the pCAGGS vector using XbaI and KpnI restriction sites, and a FLAG tag was added to the C-terminal end. Human TRIM25-V5, cloned into the pEF-IRES-puro vector, along with the RING-BB-V5, CCD-V5, and SPRY-V5 constructs, as well as the pEBG (GST-expressing) vector, GST–RIG-I(2CARD), MAVS-CARD-PRD-FLAG, FLAG-MAVS, and untagged IAV NS1 and NS1-FLAG have previously been described (18, 44). NiV-V–FLAG and TBK1-FLAG were previously described (80, 81). pEF-Bos-FLAG-MDA5 has also been described (82). cGAS 3×-FLAG, STING-FLAG, and IRF3-5D–FLAG were kindly provided by Jae U. Jung (University of Southern California). All constructs were sequenced to verify that the original sequences were correct.

Recombinant IFN-α2 (PBL Assay Science catalog no. 11100-1) and poly(dA·dT)/LyoVec (InvivoGen catalog no. tlrl-patn) were used according to the manufacturers’ instructions. Rabies virus leader sequence RNA (RABV_LE_) was generated by *in vitro* transcription as previously described (28).

### Pulldowns, coimmunoprecipitations, and immunoblotting.

Procedures for GST and FLAG pulldown assays were performed as previously described (18, 33). Briefly, approximately 48 h after transfection with the indicated GST-fused or FLAG-tagged plasmids, HEK 293T cells were centrifuged and lysed in NP-40 buffer (50 mM HEPES [pH 7.4], 150 mM NaCl, 1% [vol/vol] NP-40, and protease inhibitor cocktail [Sigma catalog no. P2714]). Whole-cell lysates were centrifuged at 17,000 × *g* for 20 min at 4°C and then mixed with a 50% slurry of glutathione-conjugated Sepharose beads (Amersham Biosciences) or anti-FLAG M2 FLAG beads (Sigma-Aldrich). The lysates were incubated with beads for 4 h at 4°C, followed by extensive washing of the beads with NP-40 lysis buffer. Proteins were eluted with Laemmli SDS loading buffer by boiling suspensions for 5 min at 95°C.

For coimmunoprecipitations, HEK 293T cells were lysed in NP-40 buffer or radioimmunoprecipitation assay (RIPA) buffer (50 mM Tris-HCl [pH 7.4], 150 mM NaCl, 1% [vol/vol] NP-40, 0.5% [wt/vol] deoxycholic acid, 0.1% [vol/vol] SDS, and protease inhibitor cocktail [Sigma catalog no. P2714]) in the case of experiments assaying polyubiquitination. WCLs were incubated with 0.5 to 2.5 mg of antibody at 4°C overnight, followed by incubation with a 50% slurry of protein A/G agarose (Pierce catalog no. 20422) for 2 h at 4°C.

Immunoblot analysis was performed as previously described (33). Briefly, proteins were resolved by Bis-Tris polyacrylamide gel electrophoresis (PAGE) and transferred onto polyvinylidene difluoride (PVDF) membranes (Bio-Rad). Membranes were blocked with 5% (wt/vol) nonfat dry milk in PBS-Tween 20 (PBS-T) for 1 h, followed by probing with primary antibodies at room temperature for 1 h or at 4°C overnight.

The following primary antibodies were used at a 1:2,000 dilution: anti-FLAG (M2; Sigma), anti-GST (GST-2; Sigma), anti-TRIM25 (2/EFP; BD Biosciences), polyclonal anti-SeV (PD029; MBL), and anti-p97 (clone 18, VCP; BD Biosciences). The following primary antibodies were used at a 1:1,000 dilution: anti-ISG15 (F-9; Santa Cruz Biotechnology), polyclonal anti-ISG54 (12604-1-AP; Proteintech), anti-RIG-I (Alme-1; AdipoGen), polyclonal anti-MAVS (Cell Signaling; 3993S); anti-IFI16 (D8B5T; Cell Signaling Technology), polyclonal anti-ZAP (ZCCHV) (ab154680; Abcam), anti-tAg (Pab2003; kindly provided by Ellen Cahir-McFarland [Biogen, Cambridge, MA]), polyclonal anti-cGAS (HPA031700; Sigma), anti-STING (clone 723505; R&D Systems), anti-TBK1 (D1B4; Cell Signaling), and anti-NS1 (kindly provided by Adolfo Garcia-Sastre, Icahn Medical School at Mount Sinai) (83). Anti-β-actin (AC-15; Sigma) was used at a 1:10,000 dilution. Anti-V5 (R960-25; Novex) was used at a 1:5,000 dilution. Antiubiquitin (P4D1; Santa Cruz Biotechnology) was used at a 1:500 dilution.

Secondary anti-mouse or anti-rabbit IgG-horseradish peroxidase (HRP) antibodies (7074S and 7076S; Cell Signaling) were used at a 1:2,500 dilution and incubated at room temperature for 1 h. Protein bands were visualized with the enhanced SuperSignal West pico or femto chemiluminescence reagent (Thermo Fisher; catalog no. 34578 and 34096) and were detected by a luminescent imaging system (Fuji LAS-4000).

### Mass spectrometry.

For the identification of cellular interacting proteins of the JCV tAg, large-scale FLAG pulldown was performed by transfecting 10- by 10-cm dishes of HEK 293T cells with 20 μg of DNA per dish. Cells were harvested and resuspended in ice-cold PBS, followed by lysis in NP-40 buffer (50 mM HEPES [pH 7.4], 150 mM NaCl, 1% [vol/vol] NP-40, and protease inhibitor cocktail [Sigma; P2714]) and preclearing of cell lysates using Sepharose beads for 2 h at 4°C. Precipitates were extensively washed with 1% NP-40 lysis buffer, and proteins bound to M2 FLAG beads were separated on NuPAGE 4 to 12% Bis-Tris gradient gel (Invitrogen). The gel was silver stained according to the manufacturer’s instructions (SilverQuest kit; Invitrogen), and bands specific to tAg-transfected lanes, but not the vector control, were excised and analyzed by ion trap mass spectrometry at the Harvard Taplin Biological Mass Spectrometry Facility in Boston, MA.

### IFN-β luciferase reporter assays.

HEK 293T cells, seeded into 12-well plates (∼1 × 10^5^ cells per well), were transfected with 200 ng of the IFN-β luciferase construct and 300 ng of the β-galactosidase (β-gal)-expressing pGK-β-gal plasmid using Lipofectamine and Plus reagent (Life Technologies) as described in reference 33. Either cells were infected as indicated in the figures with SeV (10 hemagglutination units [HAU]/ml) to activate endogenous RIG-I or they were cotransfected with the following amounts of plasmids to induce activation of innate immunity: 1 ng of plasmid encoding GST–RIG-I(2CARD) or GST (negative control), 100 ng FLAG-MDA5, 100 ng cGAS-3× FLAG, and 25 ng STING-FLAG, 10 ng TBK1-HA, or 300 ng IRF3-5D–FLAG. Forty hours after transfection, cells were lysed and subjected to a luciferase assay (Promega) according to the manufacturer’s instructions. Luminescence and absorbance were measured using a BioTek Synergy HT microplate reader, and luciferase values were normalized to β-gal activity to control for transfection efficiency.

### Quantitative RT-PCR analysis.

HEK 293T cells were transfected with either an empty vector, plasmids encoding FLAG-tagged polyomavirus tAgs, or IAV NS1 (positive control), together with GST–RIG-I(2CARD) (1 ng) or IRF3-5D–FLAG (100 ng). Twenty-four hours posttransfection, total cellular RNA was extracted with the E.Z.N.A. HP total RNA kit (Omega Bio-tek). RNA quality was assessed using a NanoDrop Lite spectrophotometer. Usually, 50 to 500 ng of RNA was used for a one-step quantitative reverse transcription-PCR (qRT-PCR) using the SuperScript III Platinum one-step qRT-PCR kit with ROX dye (Life Technologies) with commercially available FAM (6-carboxyfluorescein) reporter dye primers (Integrated DNA Technologies) specific for the genes *OAS1*, *CCL5*, *IFIT2*, *IFNB1*, *IL6*, *MX1*, and *TNF.* Gene expression levels were normalized to that of cellular *GAPDH.* The fold induction of each target gene relative to values from vector-transfected cells was calculated using the comparative threshold cycle method (ΔΔ*C_T_* method). qRT-PCR assays were performed using a model 7500 Fast RT-PCR system (Applied Biosystems).

For analyzing JCV transcript expression, total RNA from JCV-infected cells was isolated as described above and then analyzed using the following primers detecting the mRNA encoding VP1 or TAg: for *VP1*, 5′-CGCCTTGTGCTCTGTGTTCAT-3′ (forward), 5′-CATGACAATGGTGCAGGGAAGCC-3′ (reverse), and 5′-/56-FAM/CAGGGGGTG/ZEN/CTTTTTAATTACAGAACAAAGTACCCAGATGGAAC/3IABkFQ/-3′ (probe), and for *TAg*, 5′-GAGAAGTGGGATGAAGACCTGTTT-3′ (forward), 5′-AGAGTGTTGGGATCCTGTGTTTT-3′ (reverse), and 5′-/56-FAM/CATCATCAC/ZEN/TGGCAAACATTTCTTCATGGCA/3IABkFQ/-3′ (probe).

### JCV infection studies.

SVGA cells were infected with JCV (Mad-1) in infection medium (MEM containing 2 mM l-glutamine and 1% [vol/vol] penicillin-streptomycin) with rocking every 15 min. After 2 h, medium was supplemented with complete MEM that contained 2% (vol/vol) heat-inactivated fetal bovine serum, 2 mM l-glutamine, and 1% (vol/vol) penicillin-streptomycin, and cells were incubated under standard culture conditions for the times indicated in the figures.

To determine the frequency of JCV-infected cells, infected SVGA cells were rinsed in PBS and then fixed using 4% (wt/vol) paraformaldehyde (Santa Cruz Biotechnology) for 30 min. Cells were then permeabilized and stained with 1 μg/ml monoclonal anti-VP1 (PAB597, raised to SV40, kindly provided by Ellen Cahir-McFarland [Biogen, Cambridge, MA]) in dilution buffer (1% BSA and 0.3% Triton X-100 in PBS) at room temperature for 1 to 2 h or at 4°C overnight. Cells were rinsed with PBS and then stained with goat anti-mouse Alexa Fluor 488 secondary antibody (Life Technologies; catalog no. A-11001) at a dilution of 1:2,000 at room temperature for 1 to 2 h. VP1-positive cells were quantified by flow cytometric analysis on an LSR-II machine, followed by analysis using the FlowJo software.

Alternatively, JCV-infected SVGA cells were harvested at the times indicated in the figures, and mRNA levels of the viral genes *VP1* and *TAg* were measured by qRT-PCR as described in detail above.

### Confocal microscopy.

SVGA cells were grown on coverslips in 12-well plates (∼3,000 cells per well), followed by infection with JCV (Mad-1) for the number of days indicated in the figures. Cells were fixed on coverslips in 4% (wt/vol) paraformaldehyde (in PBS) for 30 min, permeabilized, and then stained with anti-VP1 as described for flow cytometry analysis. Cells were rinsed with PBS, stained with goat anti-mouse Alexa Fluor 488 secondary antibody (Life Technologies; catalog no. A-11001) and again rinsed with PBS before the coverslips were mounted in Mowiol mounting medium containing DAPI (4′,6-diamidino-2-phenylindole). The slides were analyzed on an Olympus IX81 inverted widefield microscope by automatic tiling, and images shown are representative of the percentage of infected (VP1-positive) cells for a given coverslip of individual experiments.

### RNA pulldown assay.

*Lnczc3h7a* was amplified as a PCR product using the primers 5′-TAATACGACTCACTATAGGGAGAATACGTAATTTTTAAGC-3′ and 5′-GTTTGTTTGTTTGTTTGTTTTTCCAGACAGGGTTTCTCTGTATAG-3′ and then directly *in vitro* transcribed using the MEGAscript kit (Life Technologies). *In vitro*-transcribed *Lnczc3h7a* or total RNA isolated from JCV-infected SVGA cells at day 8 postinfection (termed Bio-RNA [JCV-infected]) was biotinylated using the Pierce RNA 3′ end biotinylation kit (ThermoFisher) according to the manufacturer’s instructions. HEK 293T cells were transfected with TRIM25-V5 and tAg-FLAG or TAg-FLAG for 24 h. Cells were harvested and lysed with NT2 buffer (50 mM Tris-HCl, pH 7.0, 150 mM NaCl, 1 mM MgCl_2_, 0.05% [vol/vol] NP-40, SUPERase·In [Thermo Fisher], protease inhibitor, and Ser/Thr phosphatase inhibitor). Cell lysates were incubated with *in vitro*-transcribed biotin-labeled *Lnczc3h7a* or Bio-RNA (JCV infected) for 3 h at 4°C, followed by incubation with Dynabeads M-280 streptavidin (ThermoFisher) for another 3 h at 4°C. Beads were washed several times with NT2 buffer, boiled for 10 min at 95°C, and then subjected to immunoblot analysis as indicated.

### Statistical analysis.

Statistical analyses were performed by two-tailed Student’s *t* test. A *P* of <0.05 was considered statistically significant.
