# Supplementary figures and images for: The Small t Antigen of JC Virus Antagonizes RIG-I-Mediated Innate Immunity by Inhibiting TRIM25’s RNA Binding Ability
Source: mBio. 2021 Apr 13;12(2):e00620-21. doi: 10.1128/mBio.00620-21 (PMC8092259; doi:10.1128/mBio.00620-21)

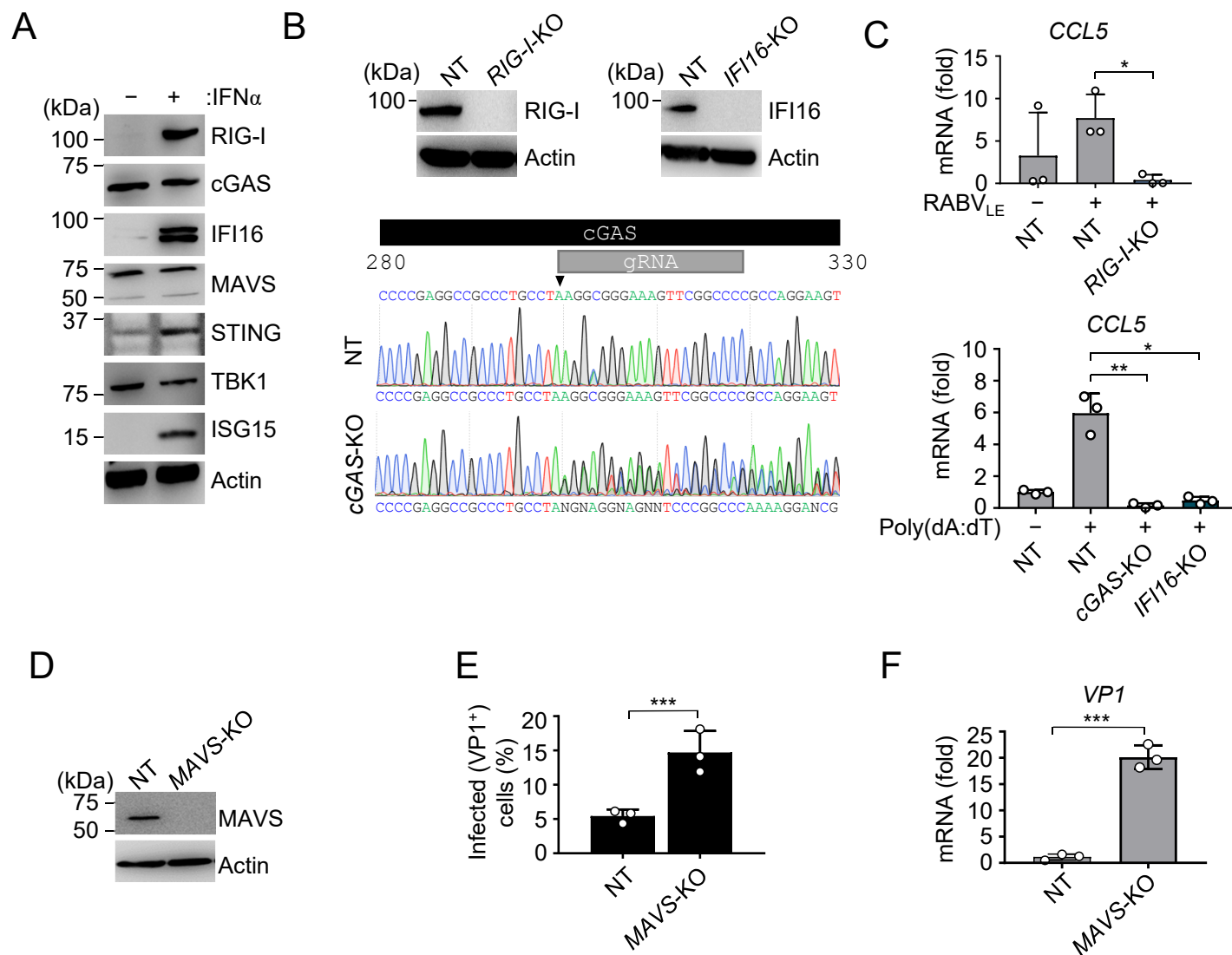

FIGURE S1

Supplement: FIG S1 [file mBio.00620-21-sf001.pdf]

A

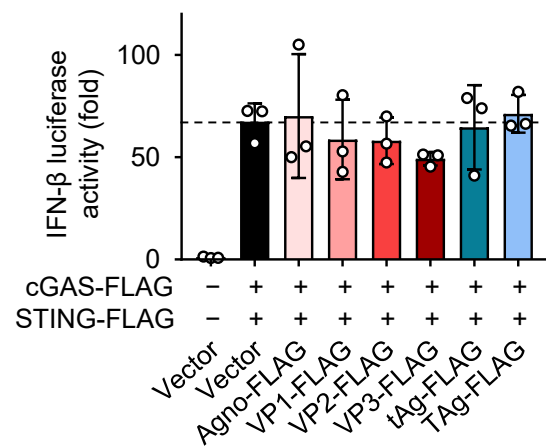

B

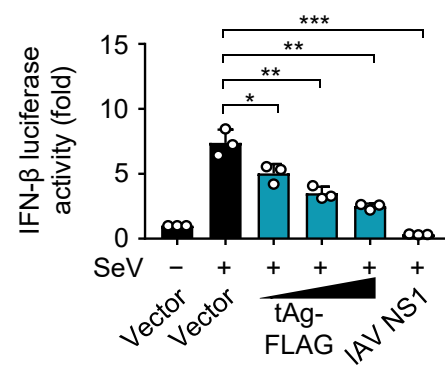

C

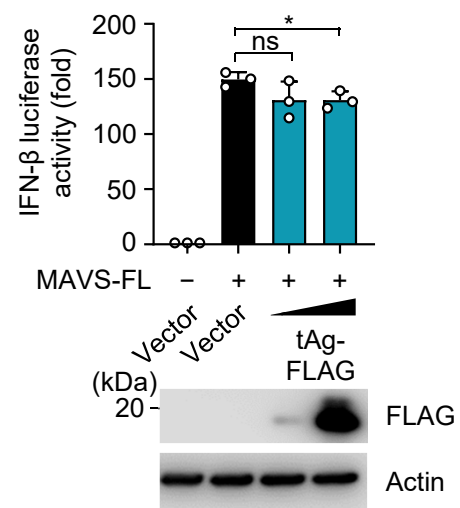

D

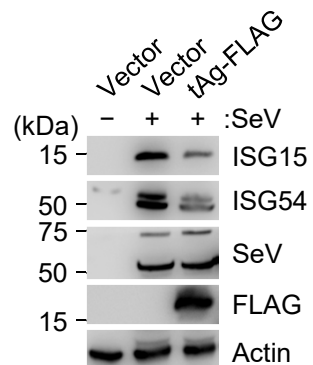

FIGURE S2

Supplement: FIG S2 [file mBio.00620-21-sf002.pdf]

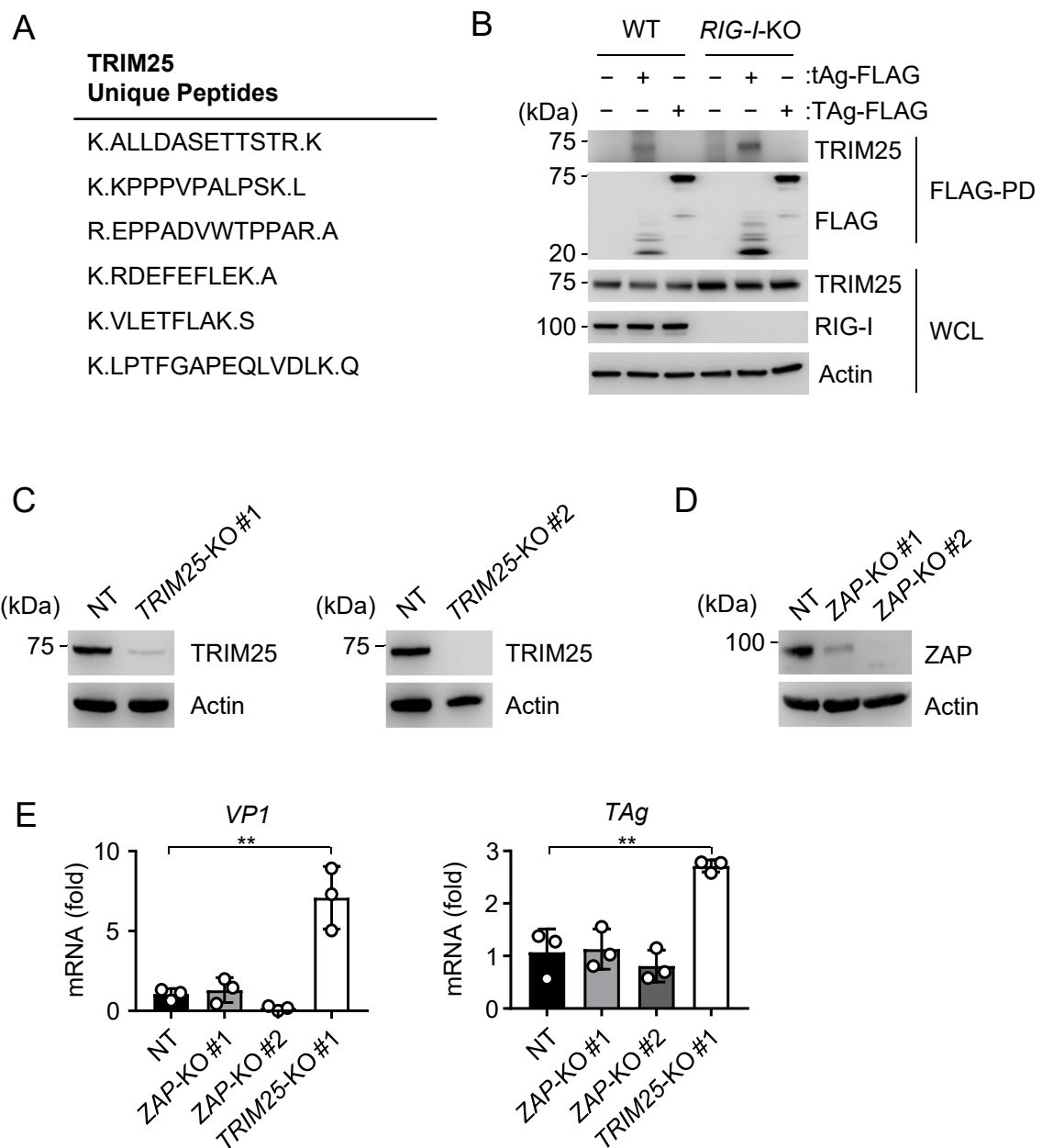

FIGURE S3

Supplement: FIG S3 [file mBio.00620-21-sf003.pdf]

A

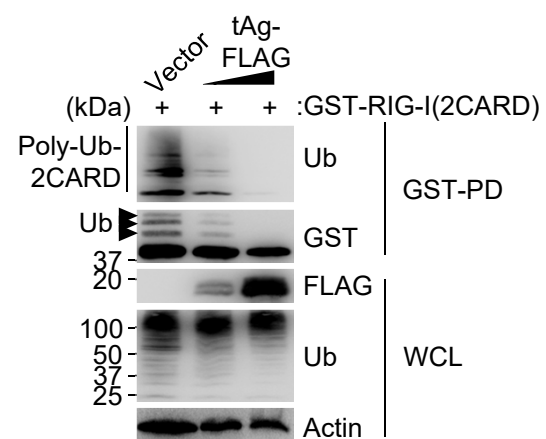

B

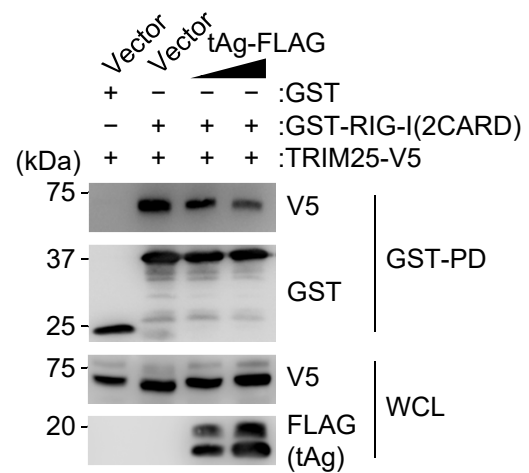

FIGURE S4

Supplement: FIG S4 [file mBio.00620-21-sf004.pdf]
