# Supplementary material for: The Small t Antigen of JC Virus Antagonizes RIG-I-Mediated Innate Immunity by Inhibiting TRIM25’s RNA Binding Ability
Source: mBio. 2021 Apr 13;12(2):e00620-21. doi: 10.1128/mBio.00620-21 (PMC8092259; doi:10.1128/mBio.00620-21)
Supplement: FIG S5 [file mBio.00620-21-sf005.pdf]

A

```
MCV     mdlvlnrkerealckllleispncygniplmkaafkrsclkhhpdkggnpvimmelntlws   60
SV40    mdkvlnreeslqlmdllglersawgniplmrkaylkkckefhpdkggdeekmkkmntlyk   60
JCV     mdkvlnreesmelmdllgldrsawgnipvmrkaylkkckelhpdkggdedkmkrmnflyk   60
BKV     mdkvlnreesmelmdllgleraawgnlplmrkaylrkckefhpdkggdedkmkrmntlyk   60
        ** ****:*   * .** :.  .:**:*:*: *: :.* : ******:   *  .:* *:.

MCV     kfqqnihklr-sdfsmfdevstkfpweeygtlkdymqsgynarfcrg-pgcmlkqlrdsk   118
SV40    kmedgvkyahqpdfggf--------wdatevfasslnpgvdamyckqwpecak--kmsan   110
JCV     kmeqggvkvahqpdfgt---------wnssev-scdfppnsdtlyckewpncat--npsvh   108
BKV     kmeqdvkvahqpdfgt---------wsssev-cadfplcpdtlyckdwpicsk--kpsvh   108
        *:::.::  :  **.           *.    .     :     :: :*:  * *       . :

MCV     caciscklsrqhcslktlkqkncltwgecfcyqcfilwfgfpptwesfdwwqktleetdy   178
SV40    cicllcllrmkhenrk-lyrkdplvwvdcycfdcfrmwfgldlcegtlllwcdiigqtty   169
JCV     cpclmcmlklrhrnrk-flrssplvwidcycfdcfrqwfgcdltqealhcwekvigdtpy   167
BKV     cpcmlcqlrlrhlnrk-flrkeplvwidcycidcftqwfgldlteetlqwwvqiigetpf   167
        * *: * *  :* . * : :.. *.* :*:* :**  ***       ::  * . : :* :

MCV     cllhlhlf   186
SV40    rdlkl---   174
JCV     rdlkl---   172
BKV     rdlkl---   172
          *:*
```

B

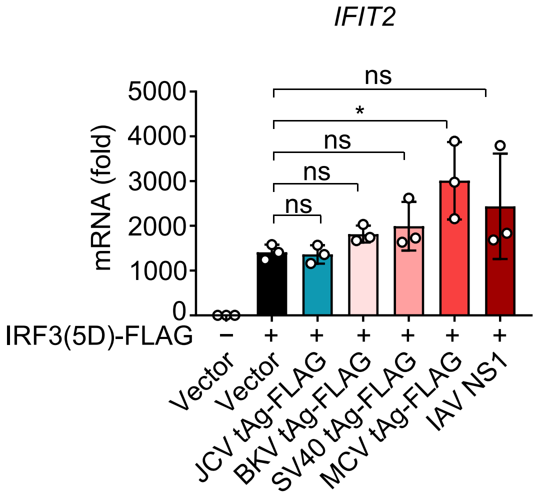

C

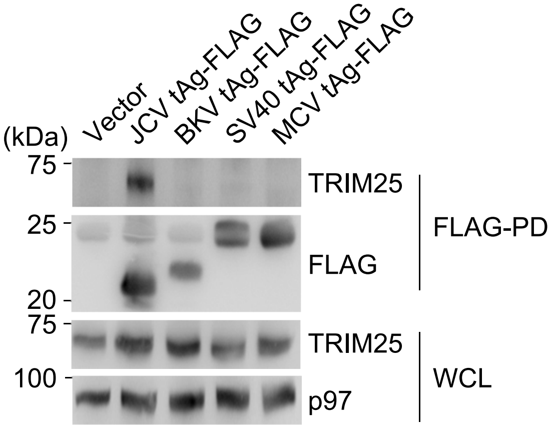

FIGURE S5
